# Chemical short-range order increases the phonon heat conductivity in a refractory high-entropy alloy

**DOI:** 10.1038/s41598-024-70500-9

**Published:** 2024-09-04

**Authors:** Geraudys Mora-Barzaga, Herbert M. Urbassek, Orlando R. Deluigi, P. Marcelo Pasinetti, Eduardo M. Bringa

**Affiliations:** 1 CONICET and Faculty of Engineering, University of Mendoza, Mendoza, 5500 Argentina; 2 Physics Department, University of Kaiserslautern-Landau, Erwin-Schrödinger-Straße, 67663 Kaiserslautern, Germany; 3 INFAP-CONICET and National University of San Luis, San Luis, 5700 Argentina; 4 Center for Applied Nanotechnology, Faculty of Sciences, Universidad Mayor, Santiago, 8580745 Chile

**Keywords:** Vibrational density of states, High-entropy alloy, Heat conductivity, Chemical short-range order, Percolation, Condensed-matter physics, Information theory and computation, Atomistic models

## Abstract

We study the effects of the chemical short-range order (SRO) on the thermal conductivity of the refractory high-entropy alloy HfNbTaTiZr using atomistic simulation. Samples with different degrees of chemical SRO are prepared by a Monte Carlo scheme. With increasing SRO, a tendency of forming HfTi and TiZr clusters is found. The phonon density of states is determined from the velocity auto-correlation function and chemical SRO modifies the high-frequency part of the phonon density of states. Lattice heat conductivity is calculated by non-equilibrium molecular dynamics simulations. The heat conductivity of the random alloy is lower than that of the segregated binary alloys. Phonon scattering by SRO precipitates might be expected to reduce scattering times and, therefore, decrease thermal conductivity. We find that, in contrast, due to the increase of the conductivity alongside SRO cluster percolation pathways, SRO increases the lattice heat conductivity by around 12 %. This is expected to be a general result, extending to other HEAs.

## Introduction

High-entropy alloys (HEAs) are a class of metals with a high configurational entropy due to the large number of elements in their composition. Given the combination of different elements with different atomic radii, the HEA lattice is intrinsically distorted^1^, which in turn influences the structure and thermal properties of the alloy. HEAs are known for their superior mechanical and thermal properties such as a good mechanical performance at different temperature, radiation resistance, and fracture toughness^2–5^.

For a number of applications, the thermal conductivity of HEAs is relevant. Thermal energy is transported by phonons, electrons, and magnons. Generally, magnon conductivity is much lower than the others and can hence be neglected. The thermal conductivity of monoatomic metals and binary/ternary alloys is mainly due to electrons. However, a recent study^6^ finds that in transition metals and transition-intermetallic compounds the phononic contributions are non-negligible.

Due to chemical disorder, the electronic contribution to heat conductivity can decrease by orders of magnitude in HEAs^7^, reaching values similar to those of phonon contribution^8^. In the kinetic theory of gases, the thermal conductivity depends on the product of the group velocity and the mean free path of the carrier. The mean free path of the carriers decreases as a result of disorder. For instance, irradiation typically leads to point defects which cause a significant decrease of thermal conductivity with radiation dose^9^. In a HEA, chemical disorder leads to a significant distortion of the crystal lattice, even in the absence of point defects or other defects. Therefore, it has been proposed that the thermal conductivity of HEAs could be adjusted by varying the composition and elemental concentration of the alloy^10^. Computational simulations are an efficient tool in the search for optimized HEAs with desired properties. An important area of application is the design of thermoelectric materials, where the reduction of the phonon lattice heat conductivity is even a design principle and has been exploited for HEAs^11^.

The phonon part of the heat conduction is of intrinsic interest for studies of keV-ion induced collision cascades in solids, since here the projectile ion imparts its energy to the atomic motion (i.e., phonons) of the target material, and electrons only provide a small contribution to the heat conduction in later phases, after electron-phonon coupling conveyed energy to the electron system^4,12,13^. Reductions in thermal conductivity would lead to the cascade staying hot longer, favoring defect recombination and lowering the final defect content, resulting in enhanced radiation resistance.

Recent studies showed that the atomic composition in HEAs need not be random on an atomic scale, but chemical effects can generate a short-range order (SRO)^14–16^. This SRO is often associated with nm-sized ordered precipitates and influences many properties of the material^17^, including mechanical^18–20^ and thermal properties^17,21–23^. The presence of several alloying elements per se implies a relatively low phonon thermal conductivity^24,25^, but the presence of precipitates would generally be expected to reduce thermal conductivity even further^25^.

Although chemical complexity has been emphasized as an important factor in reducing thermal conductivity,^26^, studies of the phonon thermal conductivity of concentrated alloys are relatively rare, in contrast to elemental materials and binary alloys^6,27,28^. A notable exception is the model study by Caro *et al.*^10^ which showed how changes in atomic mass and force constants decrease the thermal conductivity of fcc binary alloys – and also fcc multi-component alloys – considerably below that of the pure elements. Cheng *et al.*^29^ investigated the phonon conductivity of fcc CrFeCoNiCu alloys as a function of temperature and found that it deviates increasingly strongly from that of elemental Cu with decreasing temperature. Jin *et al.*^30^ discussed the contributions of electrons and phonons to the heat conductivity in HEAs for the special example of fcc-structured Ni-based alloys and demonstrate that, in particular at low temperatures, phonons dominate the heat conductivity. Samples of (FeNiCrCo)$_{1-x}$Al$_x$ have been studied as a function of Al content and temperature, but considering random samples, without the appropriate SRO that arises from the effective repulsion between Al-Al pairs^31^. Samples of (FeNiCrCo)$_{0.7}$Al$_{0.3}$ were simulated without SRO, but compared satisfactorily with experiments^32^. DFT simulation of the elastic constants coupled to analytical models to obtain the thermal conductivity was used to study 12 different bcc HEAs, finding that the relative phonon contribution can vary significantly^33^. Another ab-initio study found various behaviors in the contribution of electrons to the thermal conductivity of bcc HEAs, while the phonon conductivity was estimated with an analytical model requiring the Debye temperature^34^. Finally, experiments on both thermal conductivity and electrical resistivity were used to estimate the phonon contributions to thermal transport for several bcc HEAs below 200 K^35^. All the simulation studies above were for ‘perfect’ bulk crystals, without defects. Finally, a recent study^36^ investigated the effect of mass disorder on the thermal phonon conductivity, and found that correlations in the spatial mass distribution, i.e., mass SRO, decreased the heat conductivity.

Alternative approaches help the study of defective samples. Recently, the topology of a disordered network was related to its thermal conductivity^37^, including the generation of a dataset for prediction and tuning thermal transport. On the other hand, percolation theory has been applied to a variety of phenomena in physics, chemistry, biology, and materials science, where connectivity and clustering play an important role^38,39^. This theory has also provided insights into the behavior of more complicated models exhibiting phase transitions and critical phenomena. A recent work in this line is Ref.^40^ where the authors study the percolative phase transitions of a NbMoTaW HEA thin film during the process of filling the lattice on interstitial sites with small non-metal atoms, O or N. The phenomenon is explained by transforming a multi-color percolation into a two-color classical percolation problem where clusters with high and low electrical resistivity eventually form a percolation path contributing to a sudden reduction of the resistivity. They find a critical concentration of the conductive clusters similar to the percolative thresholds of the fcc and amorphous lattices.

Available analytical approaches to predict the phonon thermal conductivity of HEAs such as in Ref.^33,41^ are not able to include the chemical SRO. SRO in refractory HEAs is complex, and still not fully understood^23^. The low thermal conductivity of HfNbTaTiZr is desirable for thermal insulation technologies^42^. The low-temperature thermal conductivity, from nearly 0 K to 200 K was measured recently alongside the electrical resistivity, leading to estimated values of the phonon thermal conductivity similar to the ones due to the electronic thermal conductivity^35^. For high temperatures, there are measurements for a sample with 1 wt.% oxygen, giving a relatively low conductivity in the range 10–17 W/(Km) at 300–1200 K^42^.

In the present study, we focus on the effect of chemical SRO on the vibrational density of states and the phonon heat conductivity in a prototypical refractory HEA, HfNbTaTiZr, which crystallizes in the bcc structure^3,43,44^. Using an atomistic model of this alloy, we are able to study, besides the random alloy, various degrees of chemical SRO in the alloy. As a result, we show that chemical SRO can modify the heat conductivity in a sizable way.

## Methods

In this section, the main methods used for performing and analyzing the simulations in this study are presented. These are the preparation of the random HfNbTaTiZr sample, the creation of SRO, methods for the sample analysis, models to assess the percolation of clusters in the sample, the calculation of the phonon density of states, and finally the calculation of the heat conductivity.

The molecular dynamics (MD) simulations are performed with the LAMMPS code^45^. We use the embedded-atom-method (EAM) type potential by Xu *et al.*^46^. This potential was developed explicitly in order to describe the short-range order in HfNbTaTi-based quinary refractory multi-principal element alloys such as we employ in the present study.

### Sample preparation

As a first step, we create a random HfNbTaTiZr alloy. The single-crystalline body-centered cubic (bcc) sample has a cubic shape with {100} faces and an edge length of around 13.5 nm (40 lattice constants); it contains 128 000 atoms. We simulate this specimen using periodic boundary conditions.

In the random crystal, the atoms are distributed randomly over the lattice points with equal fractions of 20%. After relaxation to a final temperature of around 10 mK and low stress components around $10^{-4}$ GPa, it has a lattice constant of 3.389 Å, in good agreement with experimental data^43,47,48^.

### SRO samples

The unrelaxed random sample was evolved using a Metropolis Monte Carlo (MC), with Kawasaki-type evolution, such that atoms are swapped^49,50^. The temperature for the Metropolis rejection factor was 100 K. 10 different swaps were attempted per MC step, one for each possible chemical pair AB, with A different from B. A similar scheme was recently employed to evolve a similar sample later studied under nanoindentation^51^. The simulation was run for 4.7e6 steps. The evolution of the potential energy during the Monte Carlo run is shown in the Supplementary Material (SM), Fig. S17, and demonstrates that after 4.7e6 steps, the sample has nearly reached equilibrium.

SRO is usually quantified using the Warren-Cowley (WC) parameters^52,53^,

1 $$\begin{aligned} \alpha _{ij} = 1 - \frac{p_{ij}}{c_j} , \end{aligned}$$

to quantify the chemical SRO. Here, $p_{ij}$ is the probability of finding an atom of type *j* in the first-neighbor shell of an atom of type *i* and $c_j$ is the (average) concentration of atoms of type *j*. Thus, for a random alloy it is $\alpha _{ij} =0$; $\alpha _{ij} > 0$ characterizes atomic repulsion and while $\alpha _{ij} <0$ denotes attraction.

At this low temperature, the chemical SRO of the alloy increases with MC steps until reaching a “steady” state, according to the evolution of both the potential energy and the Warren-Cowley parameters, at few million MC steps. Therefore, in what follows, the level of SRO is directly associated to the MC step number.

The WC parameters provide likely segregation pairs, but do not provide information about the structure of the aggregation. For bcc crystals, a common structure arising due to SRO is the B2 structure^23^, where an equiatomic binary alloy accommodates one chemical type in the center of the cube and the other type in the vertices.

Recent simulations of MC evolution of the HfNbTaTiZr HEA show the rise of such B2 structures for the most negative WC parameters^51^. In a B2 structure made with atoms A and B, atoms of type A only have neighbors of type B and vice versa. Therefore, to obtain clusters for a given B2 structure we select only atoms of type A which are linked to type B at the nearest neighbor distance, using the *Create bonds* tool in OVITO. Interconnected atoms form clusters and their cluster size can also be obtained with OVITO.

### Sample analysis

Atomistic snapshots are generated with OVITO^54^. OVITO was also employed to obtain coordination, clusters, and solid volume fraction of selected clusters.

The volume fraction of SRO clusters can be evaluated in different manners. It can be obtained assuming that all atoms occupy the same volume in the regular bcc lattice used for the SRO MC simulations. Then the volume fraction is given by $N_a / N_t$, where $N_a$ is the total number of atoms in the cluster, and $N_t$ is the total number of atoms in the whole sample. An alternative estimation could be obtained from the *ConstructSurfaceMesh* tool^54^ in OVITO. In this method, a virtual probe sphere of radius *r* is rolled over the cluster surface to define a smooth geometric surface, and the volume inside that surface is calculated based on Delaunay tessellation of that volume. A spherical probe size of 0.29 nm is used, unless otherwise noted. For this case, to distinguish from the previous estimations using atom numbers, volume fractions are termed as $c_n$, where $n=AB$ refers to clusters the binary B2 alloy with atom types *A* and *B*.

SRO clusters are irregular. We could consider them as formed by interconnected filaments, and the freeware software *FoamExplorer*^55^ was employed to obtain an estimate of the size of those filaments. The wording “filament” is borrowed from bicontinuous foams, which form a single connected porous cluster, formed by interconnected filaments, and FoamExplorer has been used to characterize such structures. *FoamExplorer* works in the following way: surface atoms are identified according to their coordination, and then a linear “chord” is obtained, going from one surface atom to another, and the different chord lengths generate a histogram which represents the filament size distribution. A nanowire will give a very narrow histogram with the diameter of the nanowire, but irregular filaments will give some wider distributions. The following parameters were employed for FoamExplorer: for external (surface) atoms, a cutoff of 0.3 nm and a maximum coordination of 7. For internal atoms, a cutoff of 0.3 nm for first neighbors, cutoff of 0.49 nm for third neighbors, and maximum coordination at third neighbors of 26 were used.

### Percolation theory and models

Usually, the percolation model in a lattice is classified into two categories, namely, the site model and the bond model. The percolation transition is a geometrical phase transition where the critical concentration of sites (or bonds) separates a phase of finite clusters from a phase where a macroscopic, spanning, or infinite cluster (in the thermodynamic limit) is present^38^. In our case we will assume a 3D site percolation model, considering that all atomic species occupy the same volume and that they are arranged on a regular bcc lattice.

There are five atomic species involved (multicolor percolation), however, as we are interested in studying the conductivity given by the HfTi association in a B2 structure, this association will be considered as the conductive (white) species, while any other species as non-conductive (black), reducing the problem to a two-color percolation^40^.

The substrate is initially generated completely at random. Then, a Kawasaki MC simulation brings the system closer to a condition of thermodynamic equilibrium, increasing the SRO of the system. This equilibrium is given at a certain temperature and keeping constant the number of atoms of each species (canonical ensemble). Therefore, this is not a strictly classical random percolation involving a random sequential adsorption process, which is essentially an out-of-equilibrium process, where each site has the same probability *p* of being occupied. Instead, this is a sort of thermal percolation, as in Giménez *et al.*^56^, where the probability for each site of being occupied depends on the temperature and the potential used in the Kawasaki exchange process. In our case, the percolation threshold, $p_c$, will be associated with the number of MC steps necessary for the formation of a percolating cluster. It will be shown that the relevant percolating cluster in this case is HfTi with B2 structure.

At the percolation transition, the cluster size distribution of the system, *n*(*s*), is expected to have a power law behavior^38^,

2 $$\begin{aligned} n(s) \propto s^{-\tau } , \end{aligned}$$

where $\tau$ is known as the Fisher exponent, taking the value $\tau \approx 2.19$ for site percolation in the bcc lattice.

### Phonon density of states

Previous research has underscored the significance of the vibrational density of states (VDOS) in evaluating the thermal characteristics of diverse nanostructured materials^57–61^. Molecular dynamics simulation allows to calculate easily the velocity autocorrelation function

3 $$\begin{aligned} Z(t)=\frac{1}{N} \sum _{i=1}^{N}\frac{{\varvec{v}}_i(t) \cdot {\varvec{v}}_i(0)}{{\varvec{v}}_i(0) \cdot {\varvec{v}}_i(0)} , \end{aligned}$$

where ${\varvec{v}}_i(t)$ denotes the velocity vector of atom *i* at time *t*, and the summation is performed over all atoms within the system. The VDOS is then given by the Fourier transformation,

4 $$\begin{aligned} G(\omega )=\int _{0}^{\infty } Z(t) \cos (\omega t) \, dt . \end{aligned}$$

To obtain the dependency on the wavelength $\lambda$, the approximate relation $\lambda =v_s/\omega$ can be used, where the sound velocity $v_s$= 3.67 km/s can be obtained as $v_s=\sqrt{B/\rho }$, using the bulk modulus *B* = 134 GPa and the mass density $\rho$ = 9.93 gcm$^{-3}$ of the HfNbTaTiZr HEA as obtained by Alhafez *et al.*^51^.

### Heat conductivity

There are several approaches to calculate the thermal conductivity^62^. For instance, both equilibrium and non-equilibrium approaches were shown to give similar results for amorphous C^63^. In this study, we took the approach of non-equilibrium molecular dynamics, that has frequently been used to calculate the thermal conductivity $\kappa$^10,64^. It obtains the thermal conductivity from Fourier’s law:

5 $$\begin{aligned} \kappa =J \frac{\Delta z}{ \Delta T } , \end{aligned}$$

where *J* is the heat flux and $\Delta T/\Delta z$ the temperature gradient across the system.

In detail, first a Langevin thermostat is applied for 0.1 ns to the entire crystal, so that it reaches the target temperature $T=300$ K. Then, two regions at opposite boundaries of the HEA, 0.7 nm thick, are fixed during the rest of the simulation. Adjacent to these fixed regions, the atoms in a strip of width 0.7 nm are subjected to Langevin thermostats to keep their temperatures at 0.8*T* and 1.2*T*, respectively. This induces a thermal gradient, ${\Delta T}/{ \Delta z }$ in the system, which is applied for 0.6 ns. During the following 0.4 ns the thermodynamic properties of the system are calculated every 0.5 ps. This is in particular the heat *Q* which is calculated as the average between the energy added and subtracted to the hot and cold thermostat, respectively; the heat flux *J* is determined from *Q* by dividing by the lateral cross section of the crystal and the time during which the heat is delivered.

## Results

In this section, the results of this study are presented. We discuss the chemical SRO established in the HfNbTaTiZr sample, the atomic displacements due to lattice distortion, the growth of clusters in the sample and their percolation as the SRO increases, the phonon density of states, and the thermal conductivity in the sample. Finally, models for the thermal conductivity and its temperature dependence are discussed.

### Chemical SRO in HfNbTaTiZr

**Figure 1:**
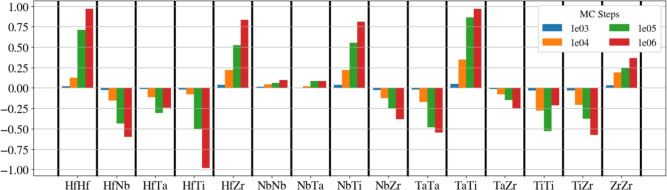
Evolution of the Warren–Cowley parameters, Eq. (1), with the number of MC steps. Only the atomic pairs showing the largest deviations from 0 are shown. After 1e3 steps, the WC parameters are nearly zero, indicating that SRO is almost negligible.

The evolution of the WC parameters with the number of MC steps is plotted in Fig. 1. Only the atomic pairs showing the largest deviations from 0 are shown. A strong tendency for attraction ($\alpha < 0$) is observed for several atom pairs, in particular for HfNb, HfTi, and TiZr; this attraction leads to the build-up of clusters ordered in the B2 structure. We note that this finding is in agreement with previous studies^46,51^. Also, Ta-Ta shows a strongly negative Warren-Cowley parameter, indicating the formation of Ta clusters. Strong positive WC parameter ($\alpha > 0$) show up for instance for Hf-Hf pairs; this demonstrates that Hf prefers not to be nearest neighbor of its own species, but rather second-nearest neighbor such as in the B2 structure. Other repelling atom pairs include Zr-Zr, Nb-Ti, Hf-Zr, and Ta-Ti.

In addition to the EAM potential^65^ used in the present study, there is an alternative modified embedded atom model (MEAM) potential for HfNbTaTiZr, developed by Huang *et al.*^15,16^. In the SM, Fig. S16, the WC parameters obtained for these two potentials are compared to DFT results^65^. The EAM potential^65^ employed here shows good agreement with DFT at “low” temperatures, identifying the same two pairs with the lowest WC, HfTi and TiZr, while MEAM provides positive WC values for those pairs^66^. We note that experiments at high temperatures, above 800 K, provide a complex picture, including TaNb-rich precipitates and HfZr-rich precipitates with a slightly different bcc phase^47,67–69^, and a HfZr-rich hexagonal phase^47,68,69^. Further studies, beyond the scope of this work, would be required to asses the applicability at high temperatures of the EAM potential used here..

### Atomic displacements due to lattice distortion

In general, large atomic displacements are related to anharmonicity and lowering of the thermal conductivity^70^. In addition to chemical disorder, we note that lattice distortion has been assigned a large role in decreasing the thermal conductivity in bcc HEAs^33^, and it can be included in simulation supercells within the Coherent Potential Approximation (CPA) method, before calculating transport properties^35^. Within the Debye approximation, thermal displacements are 0.007 nm at 300 K. For HfNbTaTiZr, an ab-initio lattice distortion leading to atomic displacements with a mean value of 0.018 nm, much larger than expected thermal values, was reported in Ref.^35^.

In the SM, we display the pair correlation function, *g*(*r*), for a perfect bcc crystal and both the random and SRO alloys, with SRO after 4.7e6 steps, after relaxation, at 300 K as Fig. S1. The width at half-maximum of the nearest-neighbor peak is 0.025 nm for random and 0.022 nm for SRO, within the range of values for other bcc HEAs^33^. As expected, SRO reduces the width of the *g*(*r*) peaks. Partial *g*(*r*) are also shown, indicating a clear narrowing of the peaks for SRO, and the formation of HfTi pairs.

We also obtain the histogram of atomic displacements, as detailed in the SM, which extends to $\sim 0.05$ nm, as in other bcc HEAs^35^. This is shown in Fig. S2a and gives mean displacement values of 0.0173 nm and 0.0153 nm, for random and SRO samples. The value for the random sample is slightly lower than the ab-initio value. SRO reduces the lattice distortion thanks to the formation of the B2 ordered structures and, therefore, this would help increasing the thermal conductivity. For NbTaTiVZr, atomic displacements were discriminated by chemical species, with Ta having the lowest value and Ti the largest one. Similar results are found here, as shown in Figs. S2b–c. Ti would be a “rattler”, i.e. the species with the largest displacements, reducing heat conductivity^70^.

A recent experimental study on Si nanoribbons found that strain gradients increase phonon scattering and significantly decrease thermal conductivity^71^. They had strains $\sim 0.05$ and strain gradients $\sim 0.1$/nm. In our HEA there are huge strain fluctuations along the sample due to size mismatch, which are akin to strain gradients. The strain and strain gradient can be roughly estimated as the root-mean-square displacement over the nearest-neighbor distance, and the nearest-neighbor distance squared, respectively, to give strains $\sim 0.06$ and strain gradients $\sim 0.2$/nm, consistent with the low thermal conductivity obtained in the simulations.

### Cluster growth and percolation

Considering that the heat flow might prefer to travel across SRO structures, it is important to analyse the evolution of the SRO clusters in detail. The more relevant results are presented here while a more detailed analysis of the cluster structure, including coordination histograms, surface-to-volume evolution and fractal dimension is presented in the SM.

**Figure 2:**
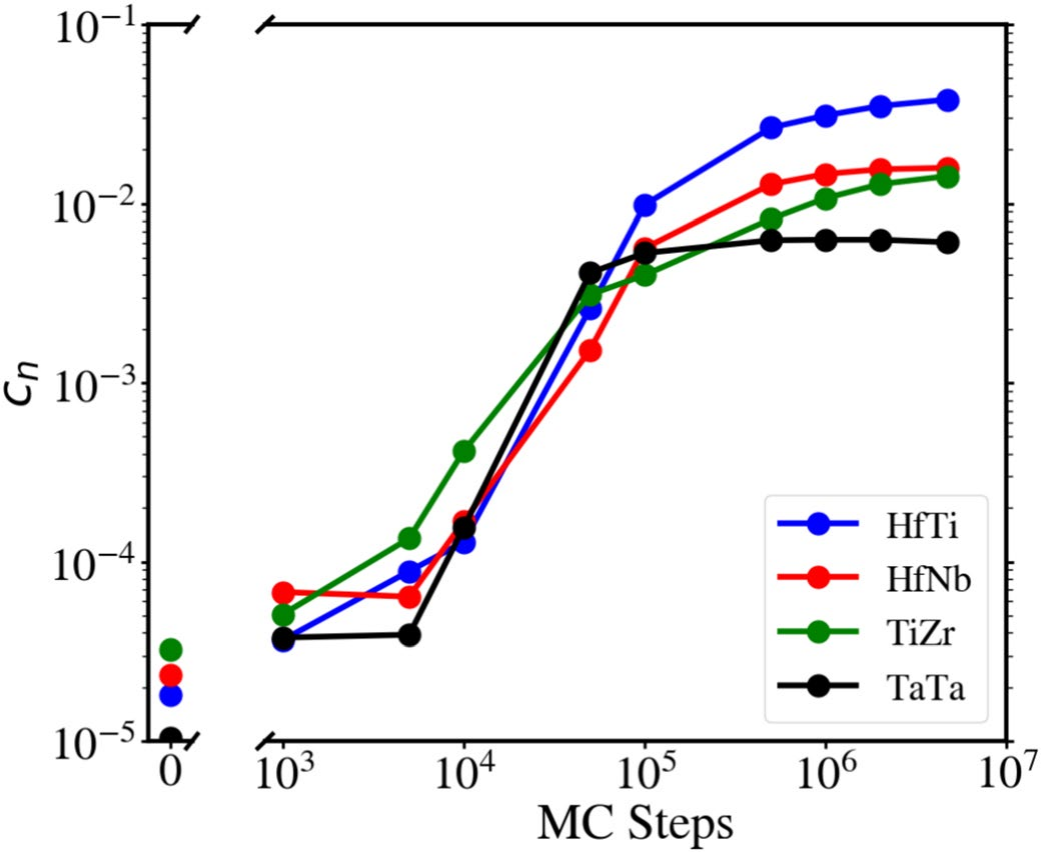
Volume fractions $c_n$ of the different B2 phases found in the short-range-ordered HEA after a certain number of MC steps, obtained with *SurfaceMesh* from OVITO^54^, for the largest clusters.

Fig. 2 presents the volume fractions *c* of the 4 relevant phases in the samples as a function of the MC steps. These have been determined using the *ConstructSurfaceMesh* tool in OVITO software^54,72^ with a probe sphere diameter of 4.0 Å. Only the volume fraction of the largest cluster was considered because, from percolation theory, the largest cluster will dominate transport across the sample. Note that these volume fractions are at most of the order of a few percent, for the last frame with the largest SRO. Their values can be found in Table S1. All these clusters display a jump in the associated volume fraction at the same time, slightly before 1e5 MC steps, which can be associated with a percolation threshold.

**Figure 3:**
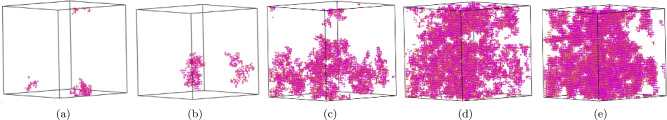
Series of snapshots illustrating the presence of B2-structured HfTi clusters in samples with the SRO established after an increasing number of MC steps: (**a**) 1 000, (**b**) 10 000, (**c**) 50 000, (**d**) 100 000, (**e**) 1 000 000. Only the largest cluster is shown in each snapshot; due to the periodic boundary conditions it may appear disconnected, but it is not.

Precipitates in alloys can be complex, with coherent or incoherent interfaces and complex geometries, including lamellar or fibrous structures^73^. We illustrate the appearance and evolution of short-range-ordered clusters for the example of HfTi in Fig. 3, which shows the largest HfTi B2 cluster at a given MC step; the corresponding snapshots for the other atom pairs discussed above are contained in the SM, Fig. S3. Similar B2 clusters, with highly irregular shape, were recently reported in simulations of the NbMoTa alloy, due to diffusive evolution^74^.

**Figure 4:**
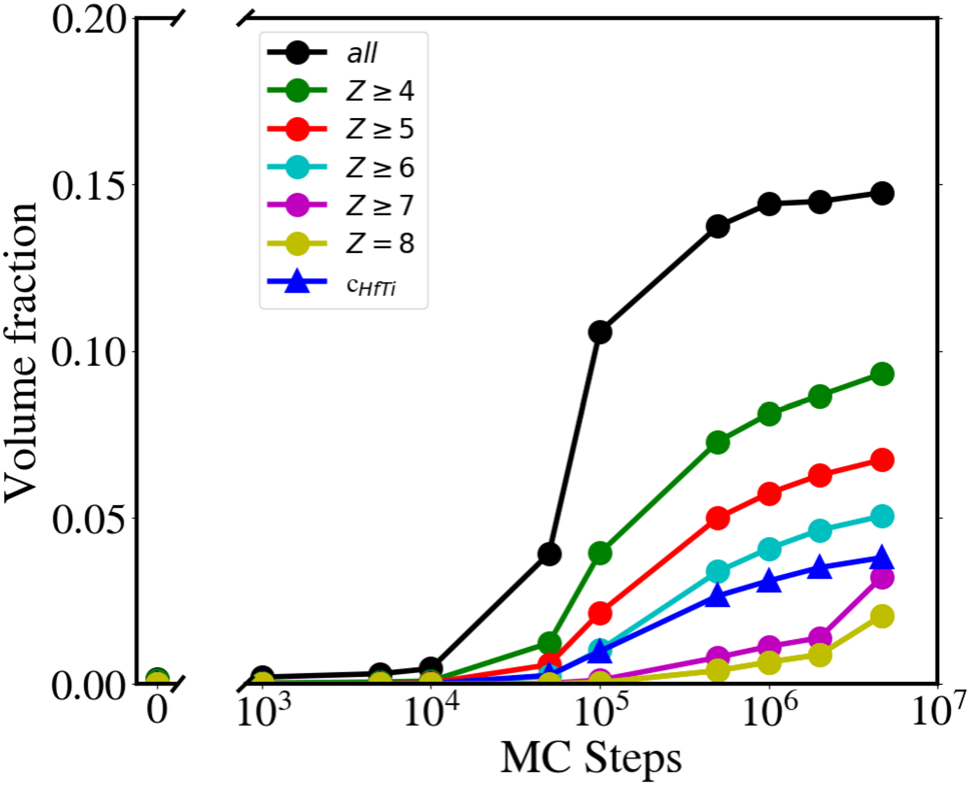
Volume fraction of the largest B2 HfTi cluster, calculated as $\hbox {N}_{a}/\hbox {N}_t$, filtering the number of atoms ($\hbox {N}_a$) according to their coordination *Z*. $Z=8$ corresponds to bulk B2 atoms. Only Hf and Ti atoms with at least one HfTi bond are included. $c_{\text{HfTi}}$, indicating the fraction obtained from *SurfaceMesh* in OVITO^54^, is also shown.

Cluster topology appears quite complex. Given the “tortuosity” of the SRO cluster shown in Fig. 3, some atoms with low coordination *Z* could be eliminated from the estimated cluster volume fraction, and this is also shown in Fig. 4. It can be seen that the volume fraction $c_{\text{HfTi}}$ estimated with *ConstructSurfaceMesh* is only slightly lower than the volume fraction determined by counting the number of atoms in the cluster and eliminating all atoms with $Z\le$6. This is equivalent to taking bulk atoms on atoms from flat surfaces, neglecting lower-coordinated surface atoms at kinks, steps, etc. The coordination histograms at different MC steps are shown in Fig. S4, with the mean coordination shown in Fig. S5.

Fig. S6a is similar to Fig. 4, but includes not only the largest cluster but all clusters containing more than 8 atoms; these are equivalent to one atom and all its bcc neighbors in a bulk sample. Fig. S6b quantifies the evolution of the distribution of cluster sizes with the number of MC steps for the example of HfTi B2 clusters. The power law size distribution is steep, with a Fisher exponent $\tau =2.5$ for low MC steps, then decreases and reaches the value expected for the bcc lattice site percolation, $\tau =2.19$^38^, after the percolation threshold, as it will be discussed later. Figure S7 shows clusters ordered by size, with a particularly strong increase in the largest size observed between 10 000 and 50 000 MC steps. This is also related to the percolation transition. Note, also, that the number of clusters shrinks from initially > 2000 to less than 200 in the sample with the largest SRO.

**Figure 5:**
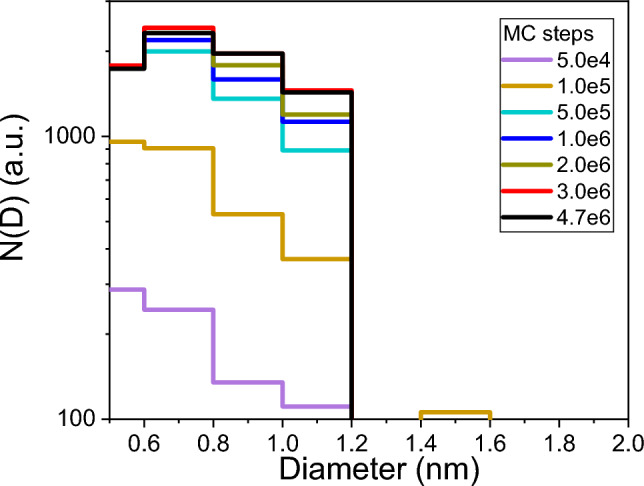
Evolution of the distribution of filament sizes, i.e., diameters *D*, for the largest HfTi cluster, for different MC steps, obtained from FoamExplorer^55^.

Fig. 5 plots the distribution of the diameter *D* of Hf-Ti filaments present in the largest HfTi cluster shown in Fig. 3, after several MC steps, as obtained by using the software FoamExplorer^55^. The figure shows that SRO features are of the order of 1 nm, in agreement with experimental studies for HEA^22,75^. Aggregation of these filaments forms the entire SRO cluster, which eventually traverses the sample. The number of filaments with that size displays a steep increase just before 50 000 MC steps, consistently with the result above.

**Figure 6:**
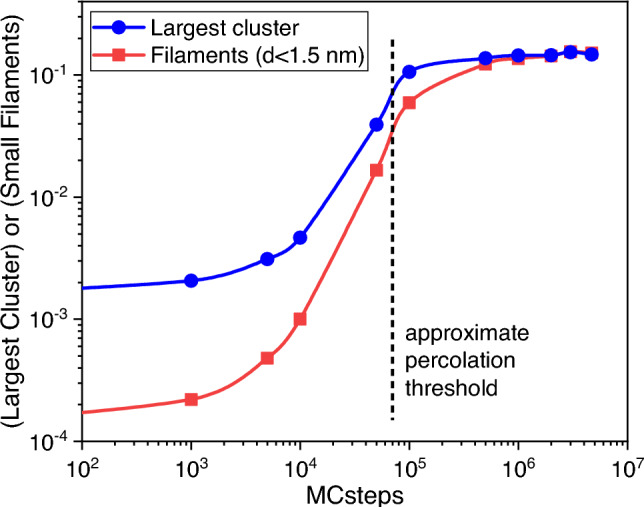
Evolution of the fraction of atoms contained in the largest HfTi cluster, together with the number of HfTi filaments with diameters smaller than 1.5 nm (arbitrary units), with the number of MC steps. Here the percolation threshold is associated with a critical number of MC steps of around $10^5$, from which a spanning cluster emerges, and it is marked by a vertical dashed line. At this threshold, just below $10^5$ steps, the number of filaments shows a sudden increase.

Fig. 6 shows the size of the largest HfTi cluster (which is also shown in the upper curve of Fig. 4) versus MC steps. This size can be interpreted as the order parameter of the percolation transition^38^. There is a clear jump and saturation above 5e4 MC steps, which can be associated with the percolation phase transition. Fig. 6 also shows that the number of filaments with diameters smaller than 1.5 nm increases with the number of MC steps, following the same trend as the cluster size. As SRO increases, there is an increase in the number of small, interconnected filaments, which is also connected to the large jump in atoms with bulk coordination, as shown in Fig. S3 in the SM.

Figs. S8–S11 display a deeper analysis of the cluster topology, including surface to volume evolution and fractal dimension estimates. Given that the fractal dimension is close to 2 for the largest SRO cluster, it can be concluded that these are not typical precipitates as found in alloys like steel.

### Vibrational density of states analysis

**Figure 7:**
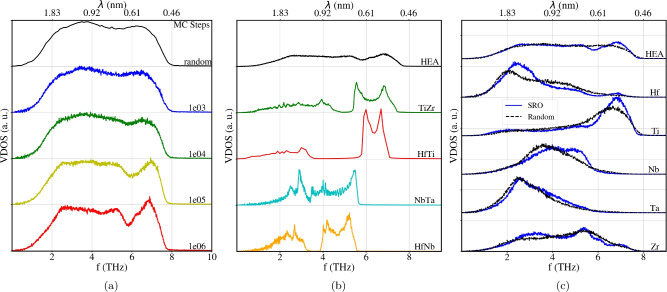
(**a**) Evolution of the VDOS for various MC steps indicated in the legend. (**b**) VDOS of the short-range-ordered alloy (after 1 million MC steps), compared to that of several binary B2 alloys. (**c**) Contribution of the constituting elements to the VDOS of the fully SRO alloy (after 1 million MC steps). In all panels, the VDOS is normalized to area 1 and shown versus frequency *f* and wavelength $\lambda$.

We now discuss the VDOS for the different alloys appearing due to SRO and the clusters described previously. The VDOS of the random alloy and its change with the number of MC steps is displayed in Fig. 7a. At low frequencies *f* (large wavelengths $\lambda$), the alloy features the universal $\propto f^2$ behavior; it is virtually unchanged by the effect of the SRO. For wavelengths above $\sim 2.5$ nm, the random alloy features a rather structureless plateau. Similar VDOS shapes, without sharp peaks, have been observed for amorphous C^63^. At smaller wavelengths $\lambda \lesssim 1$ nm, this plateau becomes affected by the SRO in that modes at around 6 THz are depleted and modes at 7 THz are populated. Beyond 8 THz, no more phonon modes exist.

A similar behavior was found when comparing the experimental VDOS for Ni versus fcc FeCoCrMnNi^76^. Our results are also consistent with ab-initio results for a 54–128-atom bcc alloy where SRO was modeled by special quasi random structures^77^. This study showed narrow peaks for the binary alloy MoTa and much broader structures for the quinary alloy MoNbTaVW. They found that the broadening in the VDOS is related to phonon broadening, caused by the reduced phonon lifetime which appears due to mass disorder and force-constant disorder in the HEA.

Fig. 7b compares the VDOS of the random alloy and of the short-range-ordered alloy after 1 million MC steps with several ordered binary alloys for comparison. HfTi, HfNb and TiZr are chosen since these compounds are found as clusters in the short-range-ordered alloys, in agreement with the WC parameters in Fig. 1. On the contrary, NbTa is an alloy which is not favored in the short-range ordering process for this interatomic potential. The B2 phase was considered for all of these binary alloys.

Clearly, the long-range ordered binaries have a strongly different VDOS than the quinary HEA. HfTi and also TiZr feature a prominent gap between the acoustic and optical phonons. In the optical part of the spectrum, the van-Hove singularities show up as strong peaks at the edges of the optical band. The comparison with the random HEA shows that the gap between acoustic and optical phonons is completely wiped out. Due to the strong lattice distortions, the structures within the optical band are destroyed and only a featureless VDOS is left over. With the onset of SRO, the highest peak of the optical band – present both in HfTi and TiZr at around 7 THz – starts to show up. The VDOS depletion in the SRO HEA around 6 THz is reminiscent of the gap between optical and acoustical phonons and is roughly situated at the corresponding gap of both HfTi and TiZr.

Finally, we display in Fig. 7c the partial (element-specific) VDOS, i.e., the contribution of each of the constituting elements to the total VDOS. It is obtained from Eq. (3) by restricting the velocity autocorrelation to the atoms of the element investigated. The sum of all partial VDOS equals the total VDOS. We observe that the high-frequency part of the VDOS (6–8 THz), which is influenced by short-range order, is populated mostly by Ti atoms, and to a lesser degree by Zr and Hf; Nb and Ta do not contribute to this frequency region. This is in agreement with our finding that the short-range order manifests mostly in the creation of HfTi and TiZr clusters, and that the features of these clusters are mirrored in the VDOS. Recently, it was found that also in the fcc HEA AlCrCoNiFe, SRO develops sharper peaks in the VDOS as compared to the random alloy^21^, in agreement with our finding for a bcc HEA.

In general, nanoscale features, like the ones for SRO, will affect thermal conductivity due to high-frequency phonons interacting with those features^25^. Figure 5 indicates that phonons with wavelength < 1.25 nm will have no problem crossing the HfTi B2 substructure after percolation occurs. This includes most relevant phonons according to the VDOS (see top axis in Fig. 7b and c).

### Lattice thermal conductivity

**Figure 8:**
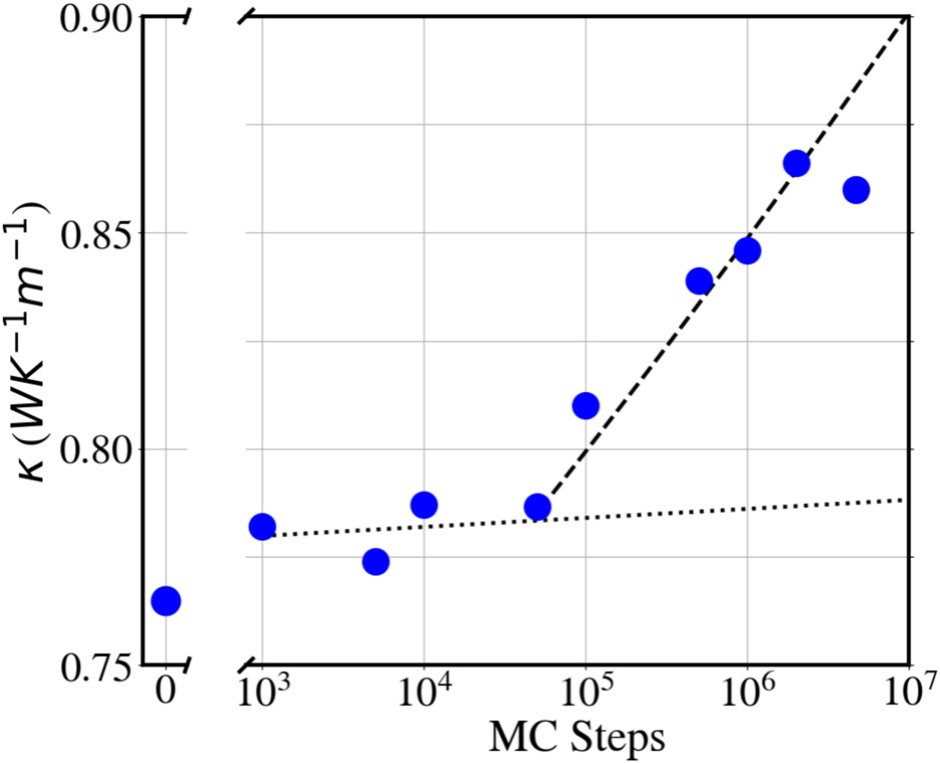
Evolution of the thermal conductivity with the number of MC steps in the SRO alloy. Lines before (dotted) and after (dashed) the percolation threshold, have been added to guide the eye.

The influence of the chemical SRO on the lattice thermal conductivity $\kappa$ (at a temperature of 300 K) is displayed in Fig. 8. An increase of $\kappa$ with SRO is observed and amounts to 12% for the case of maximum SRO, after 4.7 million MC steps. It thus appears that the effect of chemical SRO on heat conductivity should be measurable in dedicated experiments of heat conductivity. The increase in heat conductivity appears to be small as long as only small ordered clusters are present in the sample ($\le 10^4$ MC steps), but increases more strongly when large clusters – and in particular percolating clusters $\ge 10^5$ MC steps) – have been created.

There are a multitude of models for determining the effective heat conductivity in inhomogeneous media consisting of two components^78–80^, and comparison between our simulated values and models for a binary system, composed only of the random alloy matrix and a HfTi B2 phase, appear in Fig. S12, showing reasonable agreement with the Maxwell model ME-2^78^.

A more realistic model for our simulated system would include many distinct components. This is readily achieved in the ‘parallel’ model of heat conduction in which the heat flux passes through either of the media *i*,

6 $$\begin{aligned} \kappa _p = \sum _i c_i \kappa _i , \end{aligned}$$

and the ‘serial’ model in which the heat flux passes sequentially through all media *i*,

7 $$\begin{aligned} \frac{1}{\kappa _s} = \sum _i \frac{c_i}{\kappa _i} . \end{aligned}$$

Here, $c_i$ and $\kappa _i$ denote the volume fraction and the heat conductivity of medium *i*, respectively. Wang *et al.*^81^ discuss the use of the harmonic mean of these extremes,

8 $$\begin{aligned} \frac{1}{\kappa _h} = \frac{\beta }{\kappa _s} + \frac{1-\beta }{\kappa _p}, \end{aligned}$$

where the parameter $\beta$ weighs the contributions of $\kappa _s$ and $\kappa _p$ to the total conductivity. We denote this model as the ‘mixed model’ in the following.

Table 1 lists the thermal conductivities of the various individual and binary components encountered in the short-range-ordered alloy. We note that for hcp Ti, we can compare our MD value of $\kappa = 5.10$ W/(Km) with the value of 5.32 W/(Km) determined by Tong *et al.*^6^ using first-principles calculations; the agreement is satisfactory.

In order to estimate very roughly alloy effects for the random alloy, one can follow this simple approach of calculating an effective thermal conductivity using a serial or parallel model and the conductivities of all the individual elements. We obtain 5.85 or 6.24 W/(Km) for the random alloy. This is a decrease of 35% or 31% compared to pure Ta, which has the largest thermal conductivity at around 9 W/(Km). However, the decrease due to alloying effects (chemical disorder) in the random alloy has a simulated value around 0.765 W/(Km). This decrease of 91% is much larger than the estimate above, and also much larger than the maximum decrease of 70% in simulations of 4-element random alloys^10^. This can be compared to experimental results for sintered HfNbTaTiZr with 1 wt.% O content, giving 10-17 W/(Km) between 300 and 1200 K, while the parallel average gives about 35-40 W/(Km)^42^.

As SRO increases, thermal conductivity has to include different ordered B2 phases in a random matrix, and the simulated value for maximum SRO is an increase of 12% giving 0.86 W/(Km). For the model, besides the random HEA as matrix, (at least) 4 further phases are considered: HfNb, HfTi, TiZr, and Ta clusters.

**Figure 9:**
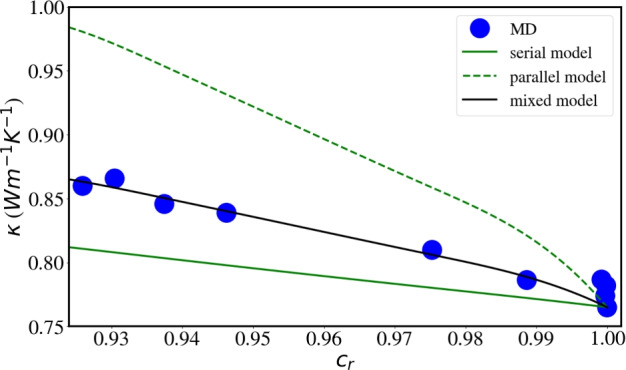
Thermal conductivity of the short-range-ordered HEA. The number of MC steps is coded in the volume fraction of the random phase, $c_r$. Simulation data are compared to the parallel, serial and mixed models of heat conduction, Eqs. (6), (7) and (8), respectively.

Fig. 9 shows the results of the parallel and serial modes of heat conduction, Eqs. (6) and (7), respectively, calculated with these values for the B2 phases. They bracket the simulated heat conductivities as the short-range-ordered HEA evolves with MC steps. The mixed model, Eq. (8), allows to describe the data accurately, using a coefficient of $\beta =0.65$. The data are plotted vs. the volume fraction of the random phase, $c_r$, which is determined from the volume fractions, $c_i$ of the 4 ordered phases considered (Fig. 2) as $c_r = 1- \sum _i c_i$. Good agreement of the mixed model, Eq. (8), with the simulation data is observed. The values for these plots, are available in the Supplementary Material (Table S1).

Recently, a somewhat similar model was proposed for homogeneous alloys with arbitrary stoichiometry, where the conductivity is evaluated as the sum over a mixture of solid solutions with integer composition, including the configurational entropy as a dividing prefactor^82^.

### Thermal conductivity from elastic properties

There are simple models of the phonon thermal conductivity, using the elastic constants to calculate the Cahill lower limit to the conductivity^83^, the Slack conductivity^84^, and the mixed-conductivity^26^. The elastic constants can be evaluated with DFT for a perfect crystal, but this presents limitations for a SRO sample^33^. The interatomic potential for HfNbTaTiZr used in this work provides excellent agreement with ab-initio elastic constants^46^, and values for both random and SRO samples were provided in Ref.^51^. All relevant parameters can be calculated from those, the lattice parameter and atomic masses.

Values for the random and SRO alloys are included in Table S2, showing almost no difference between the samples. The Grüneisen parameter is fairly high, 2.2, due to the large difference between elastic and shear moduli, as reported for many bcc HEAs^33^. Regarding the thermal conductivity, our MD results are slightly above the Cahill limit, indicating that HEAs are relatively effective as insulators. The mixed model and the Slack model values are about 7–8 times larger than the MD values. Other refractory HEAs give similar lower limits, but significantly smaller values for the other models^33^. This could be traced to the large average mass and Debye temperature for HfNbTaTiZr seen in Table S2. The VDOS for our material is far from the one for a Debye model and quantitative agreement with MD is lacking.

These analytical models do not take into account SRO except for possible elasticity changes, failing to predict an increase in conductivity due to SRO. Recent studies^33,34^ used the Slack model to estimate the phonon thermal conductivity for many HEAs, but the large discrepancy found here with MD results points to possible pitfalls in this approach.

### Temperature dependence of the thermal conductivity at high temperatures

For a model alloy with simple cubic structure, it was found that spatial correlations in the atomic mass distribution might suppress the decrease of phonon thermal conductivity with temperature for a random alloy^36^. Experiments and MD simulations of random fcc HEAs show a 50% decrease of thermal conductivity with temperature going from 300 K to 1100 K^32^.

For the case of the random alloy used here we have calculated $\kappa$ at 300 K, 1000 K, and 1300 K. The decrease at high temperatures is less than 7% compared to room temperature, unlike the calculations for the fcc HEA^32^. The sample with SRO from 4.7e6 MC steps behaves similarly, with a negligible thermal conductivity decrease with temperature. Therefore, there is a nearly constant conductivity, even for the very low level of correlated disorder in the random alloy.

The experimental thermal conductivities of individual components in HfNbTaTiZr change only moderately at high temperatures, sometimes with a slight increment^42,85^. This is different from fcc metals and alloys in the same range, typically showing some thermal conductivity decrease. Experiments for a sintered HfNbTaTiZr sample show about the same thermal conductivity for 300 K and 1000 K^42^, around 10 W/(Km).

We note that, for the time scale of the simulations, near 1 ns, the sample did not show any phase change at such a high temperature. However, there are experiments for this alloy at high temperature showing a fraction of hcp phase^86^, with calculated transition temperature values of 800 K^42^. Because of this, our simulation results at high temperatures might be considered as results only for a model single-phase bcc HEA.

Regarding collision cascades, they typically reach temperatures well above melting within a nano-sized volume^4^. The cooling time of this volume depends on the thermal conductivity at high temperatures. Therefore, it would be of interest to design HEAs not only with low conductivity, but also with a decrease in conductivity with temperature. Ab-initio simulations for several bcc HEAs do show a lowering of the electronic thermal conductivity with temperature^34^. This decrease would augment defect recombination and help radiation resistance. From this work, SRO causes an increase in the conductivity, together with possible contribution to avoid a decrease at high temperatures. From the above, this would be detrimental to radiation resistance. However, there is a competing effect, given that SRO phases might help trapping defects and facilitating their recombination. The final net outcome would have to be studied in detail for the relevant HEA.

## Discussion

Caro *et al.*^10^ simulated fcc random metallic alloys with Lennard-Jones pair potentials. They found that the thermal conductivity in random binary alloys decreases by 30–70% with respect to the conductivity of individual components, with a minimum roughly around equiatomic composition. For random 4-component alloys the decrease depends on stoichiometry, but it can be as large as 70%.

Lennard-Jones potentials were used in early studies of HEAs^10^ because the construction of reliable interatomic potentials for HEAs is challenging. Even if they work well for a random alloy, they might not offer correct results for SRO evolution. As an example, the AlFeNiCoCr potential by Farkas and Caro^87^ has been used for some SRO studies^88,89^, including conductivity simulations^21^, despite the fact that the paper describing the potential warns about the error in describing preferred formation of FeAl instead of FeNi pairs, as found in experiments and DFT simulations. For the material described in this study, there are experiments in the temperature range 750–1300 K, which find bcc precipitates rich in NbTa^67–69,90,91^, bcc precipitates rich in HfZr^91^, and hcp precipitates rich in HfZr^68,69,91^. Regarding atomistic simulations of the same alloy, using a MEAM potential, Huang *et al.*^15^ observe negative WC parameters for NbTa, TiZr, HfZr and HfTi. After MC/MD at 800 K, they observed HfTiZr-rich clusters, which gave rise to hcp precipitates; at 300 K they did not observe any hcp precipitates. Wu *et al.*^15,92^ used a charge-transfer ionic potential (CTIP) optimized with machine learning to anneal a sample above 2000 K and then cool down to room temperature. This leads to significant chemical segregation, and the most negative WC parameter is HfTi, as in our low-temperature results, without annealing. They also observe negative WC for NbTa and ZrTi pairs. Given the lack of experimental and ab-initio simulation results for SRO in this alloy at room temperature, additional studies would be required to ascertain the appropriate description of SRO evolution.

A recent analysis of conductivity in SRO samples presented the conductivity increase in terms of the Warren-Cowley parameter of the pair with lowest WC value, for an fcc HEA^21^. They used a cubic box with $21\times 21\times 21$ fcc cells (37044 atoms), which would limit the SRO cluster sizes. A similar analysis for our sample, focusing only on the pair with lowest WC parameter, which in our case is HfTi, is shown in the SM, also showing the correlation between conductivity and WC.

We note that for fcc HEAs, there are several favored pairs, as it happens for our bcc HEA (seeFig. 2) and for most HEAs. Therefore, we consider that our analysis in terms of the fraction of various B2 phases, as shown in Fig. 9, provides an improved description. We show $\alpha _{\text{HfTi}}$ versus MC step in the SM (Fig. S13), which displays a rapid increase until about 1e5 MC steps, in agreement with the observed percolation threshold, but the percolation transition is not as clear for the WC parameter as for the largest cluster size.

For the most preferred pair, HfTi, the WC parameter $\alpha _{\text{HfTi}}$ increases linearly with the fraction of B2 HfTi phase, $c_{\text{HfTi}}$, after an initial stage with negligible B2 phase, as shown in Fig. S14 of the SM. The fraction of B2 phase might be measured experimentally from diffraction studies, while the WC parameters appear as more challenging to obtain from experiments. The lattice heat conductivity $\kappa$ has a fairly linear dependence with $\alpha _{\text{HfTi}}$, as shown in Fig. S15.

Returning to bcc HEAs, there is a recent study on 12 different alloys, using ab-initio simulations to obtain their elastic constants, and models to obtain their thermal properties from those^33^. This results in thermal conductivities in the range of 0.3–1.8 W/(Km), with the exception of two quaternary HEAs with higher conductivity. For instance, HfNbTiZrV has $\kappa =0.6$ W/(Km). Using the Cahill model^83^ for the minimum thermal conductivity gives values of around 0.5 W/(Km) for all those alloys. It can be seen that the conductivity values found here are well within the expected conductivity range.

Machine learning was used to predict thermal conductivity versus temperature in alloys, based on chemical composition and a large training dataset with experimental conductivity values^93^. Such an approach would have to be enhanced for SRO alloys, given that chemical composition alone is not sufficient to determine $\kappa$.

### Percolation theory and models applied to thermal conductivity

The fact that the growth of HfTi B2 clusters with the number of MC steps is related to an increase in thermal conductivity is explained here by the presence of a percolative transition. This transition is not driven by a non-equilibrium deposition process, as is usual in percolation of RSA-type processes, but by a Kawasaki-type exchange process which is responsible for the clustering of some atomic species.

Recently, the magnetization of finite Ising systems was evaluated within the numerical *mean spin method* assuming that there is a fraction of non-magnetic atoms occupying a regular lattice, with magnetic atoms interacting with first neighbors^94^. The critical temperature for the ferromagnetic/paramagnetic transition was associated to a percolation transition of the sites occupied by magnetic atoms, i.e., above the percolation transition the average magnetization drops to zero. In our study, the increase in conductivity (magnetization) could also be related to the percolation transition for conducting/non-conducting sites.

## Summary and conclusions

By performing MD simulations on bcc single-crystalline equiatomic HfNbTaTiZr alloys with various degrees of chemical SRO, we obtained the following findings on the influence of chemical SRO on the lattice thermal conductivity.

1. Increasing chemical SRO in HfNbTaTiZr consists in the build-up of nm-sized HfTi and TiZr clusters, in agreement with the evolution of Warren-Cowley parameters.
2. Evolution of the size of the largest cluster indicates a percolation transition, which is also reflected in other metrics, including number of filaments composing the cluster, atomic coordination and fractal dimension. The cluster size distribution follows a power law, with a Fisher exponent $\tau =2.19$, as expected from site percolation in a bcc lattice^38^.
3. The cluster structure is complex, with fractal dimension around 2.4 at the transition and less than 2 at the end of our simulations, indicating a highly irregular cluster structure.
4. The percolative transition is observed in samples generated with increasing SRO through a MC Kawasaki exchange process. Therefore, this is not a strictly “classical” random percolation but a “thermal” percolation, as the one studied by Giménez *et al.*^56^. The agreement in the values of both Fisher exponent and fractal dimension at the transition, within the numerical errors, suggest, as in^56^, that the problem still belongs to the classical random percolation universality class.
5. Lattice distortion has been associated to a reduction of the thermal conductivity^33,35^. We observe that SRO leads to a 12.5% reduction of the atomic displacements due to the formation of ordered phases.
6. Thermal conductivity is related to the vibrational density of states (VDOS). In contrast to long-range chemically ordered crystals (such as B2-phase binary alloys), showing pronounced localized peaks, the VDOS of the random HfNbTaTiZr alloy is characterized by an almost structureless plateau. Acoustic and optic bands are merged, without the appearance of a gap. Van-Hove singularities, which feature band edges, are absent.
7. With increasing SRO, the VDOS of HfNbTaTiZr shows structure in the high-frequency part of the spectrum. A detailed analysis correlates this structure with the optical bands in HfTi and TiZr.
8. The MD simulated phonon thermal conductivity, $\kappa _\text{MD}$, can be compared to models which use the elastic constants for the samples. We find that $\kappa _\text{MD}$ is slightly above the Cahill lower limit^83^, but significantly lower than the estimates provided by the mixed model^26^ and the Slack model^84^.
9. Calculation of the phonon thermal conductivity from MD simulations shows a continuous increase with chemical SRO. The increase amounts to roughly 12 % above that of the random alloy and might be experimentally measurable. This increase is not related to changes in elastic constants.
10. The increase can be quantitatively explained by a simple composite model, where the material is assumed to be a combination of several phases, each with different heat conductivity. The phases correspond to the various SRO binary alloys, which have higher thermal conductivity than the random alloy matrix, leading to the net conductivity increase.
11. Conductivity does not decrease significantly with temperature for the random or SRO samples, unlike the strong decrease observed for a random fcc HEA^32^.

We note that some materials, including semiconductors like Si, include important contributions to the heat conduction by long-wavelength phonons of tens of nm^95,96^. There are relevant phonon mean-free paths of up to 1 $\mu$m, as demonstrated by experiments and simulations^97^. This leads to strong reduction of the thermal conductivity with porosity^25^, but might also lead to thermal conductivity reduction with SRO in semiconductor alloys, due to the impossibility of phonon transmission along SRO structures with a width smaller than their wavelength.

Phonon thermal conductivity will be reduced by alloy effects in most materials^24^, and precipitates within the alloy will further reduce the thermal conductivity^25^. This is because the boundary between matrix and precipitate can contribute to phonon scattering, but also because in many alloys of technological interest, precipitates have lower heat conduction that the matrix, as is the case for Si precipitates in Al alloys^73^. Despite this quite general behavior, we observe an increase in thermal conductivity in HEA with SRO, because ordered SRO precipitates have higher thermal conductivity than the random HEA matrix. We expect this behavior to be typical for any HEA with SRO, including fcc HEAs. It might be also found in other multicomponent materials with similar behavior of the thermal conductivity. In Mg alloys, addition of alloying elements usually decreases heat conductivity, but aging the alloy can lead to large thermal conductivity increments partly due to the growth of second-phase precipitates^98^. A recent study in W finds that phonon conductivity dominates over electronic conductivity for pressures above 20 GPa^99^. Some W alloys might display similar behavior and the findings in this paper might be relevant to those cases.

Besides phonons, electrons also contribute to the thermal conductivity of HEAs. Using ab-initio theory for the example of the Cantor alloy^100,101^, it has been found that – while the electron density is comparable to that of elemental metals – the electron mean-free path decreases strongly in HEAs, due to the strong lattice and chemical disorder. In the Wiedemann-Franz law the Lorenz number relates the ratio of electrical conductivity and the electronic contribution of heat conductivity to temperature^102^. Recently, the Wiedemann-Franz law was applied to experimental measurements of the electrical resistivity in HfNbTaTiZr, in order to separate phonon and electron contributions to the thermal conductivity at low temperatures^35^. Although there are many limitations to this approximate law, given a typical short mean free path for electrons in solids, we expect that electrical conductivity might also increase with SRO for HEA.

Since the alloys and phases arising with SRO have a large influence in the thermodynamic properties of HEAs, atomistic simulations, including both Monte Carlo and molecular dynamics simulations will require in the future improved potential development, including machine learning potentials, which needs to include the thermodynamics of several possible binary phases and their interfaces, in order to model SRO evolution and its effect on different properties of technological relevance, alongside machine learning approaches for improved prediction and design of thermal conductivity in complex materials^37,103^.

**Table 1:** Thermal conductivities $\kappa$ of the relevant species and B2 alloys found in the short-range-ordered HEA, as determined by MD in the present study.

Crystal	Structure	$\kappa$ [W/(Km)]
HEA(r)	BCC	0.765
HEA(SRO)	BCC	0.86
HfNb	B2	3.27
HfTi	B2	2.66
TiZr	B2	4.31
Hf	BCC	4.71
Nb	BCC	6.42
Ta	BCC	9.59
Zr	BCC	5.39
Ti	BCC	8.24
Ti	HCP	5.10

HEA(r) indicates the random alloy, while HEA(SRO) indicates the alloy after 4.7e6 MC steps

## Supplementary Information

Supplementary Information.

## Data Availability

The data that supports the findings of this study are available from the corresponding author upon reasonable request.
