# Supplementary material for: Chemical short-range order increases the phonon heat conductivity in a refractory high-entropy alloy
Source: Sci Rep. 2024 Sep 4;14:20628. doi: 10.1038/s41598-024-70500-9 (PMC11375216; doi:10.1038/s41598-024-70500-9)
Supplement: Supplementary file 1 — Supplementary Information. [file 41598_2024_70500_MOESM1_ESM.pdf]

Chemical short-range order increases the phonon heat conductivity in a refractory high-entropy alloy
Supplementary Material

Geraudys Mora-Barzaga,[1] Herbert M. Urbassek,[2,*] Orlando R. Deluigi,[1] P. Marcelo Pasinetti,[3] and Eduardo M. Bringa[1,4,†]

[1]CONICET and Facultad de Ingeniería, Universidad de Mendoza, Mendoza, 5500 Argentina
[2]Physics Department, University Kaiserslautern-Landau, Erwin-Schrödinger-Straße, D-67663 Kaiserslautern, Germany
[3]INFAP-CONICET and Universidad Nacional de San Luis, San Luis, 5700 Argentina
[4]Centro de Nanotecnología Aplicada, Facultad de Ciencias, Universidad Mayor, Santiago, Chile 8580745
(Dated: August 2, 2024)

The Supplementary Material includes additional figures and analysis.

Atomic displacements due to lattice distortion

Fig. S1 shows the pair correlation function $g(r)$ for the perfect crystal, random and SRO samples.

Fig. S2 shows the histogram of atomic displacements, for all atoms and also discriminated by chemical type, for both the random and SRO samples. We obtain this as $\mathrm{RMSD}_i = |\boldsymbol{r}_i - \boldsymbol{r}(0)_i|^2$, where $\boldsymbol{r}_i$ is the position vector of atom i in the relaxed sample and $\boldsymbol{r}(0)_i$ denotes the position of that atom in the ideal, perfect, bcc crystal. For thermally induced displacements in a harmonic potential, the distribution will peak at zero. The shifted peak observed here indicates strong anharmonicity which is partly due to the cocktail effect in HEA.

SRO cluster evolution

Table S1 shows the fraction of all the relevant clusters, together with the resulting heat conductivity, versus MC step.

Fig. S3 expands Fig. 2 of the main text and shows the evolution of several important clusters in the sample during increasing SRO.

*Electronic address: urbassek@rhrk.uni-kl.de; URL: http://www.physik.uni-kl.de/urbassek/
†Electronic address: ebringa@yahoo.com

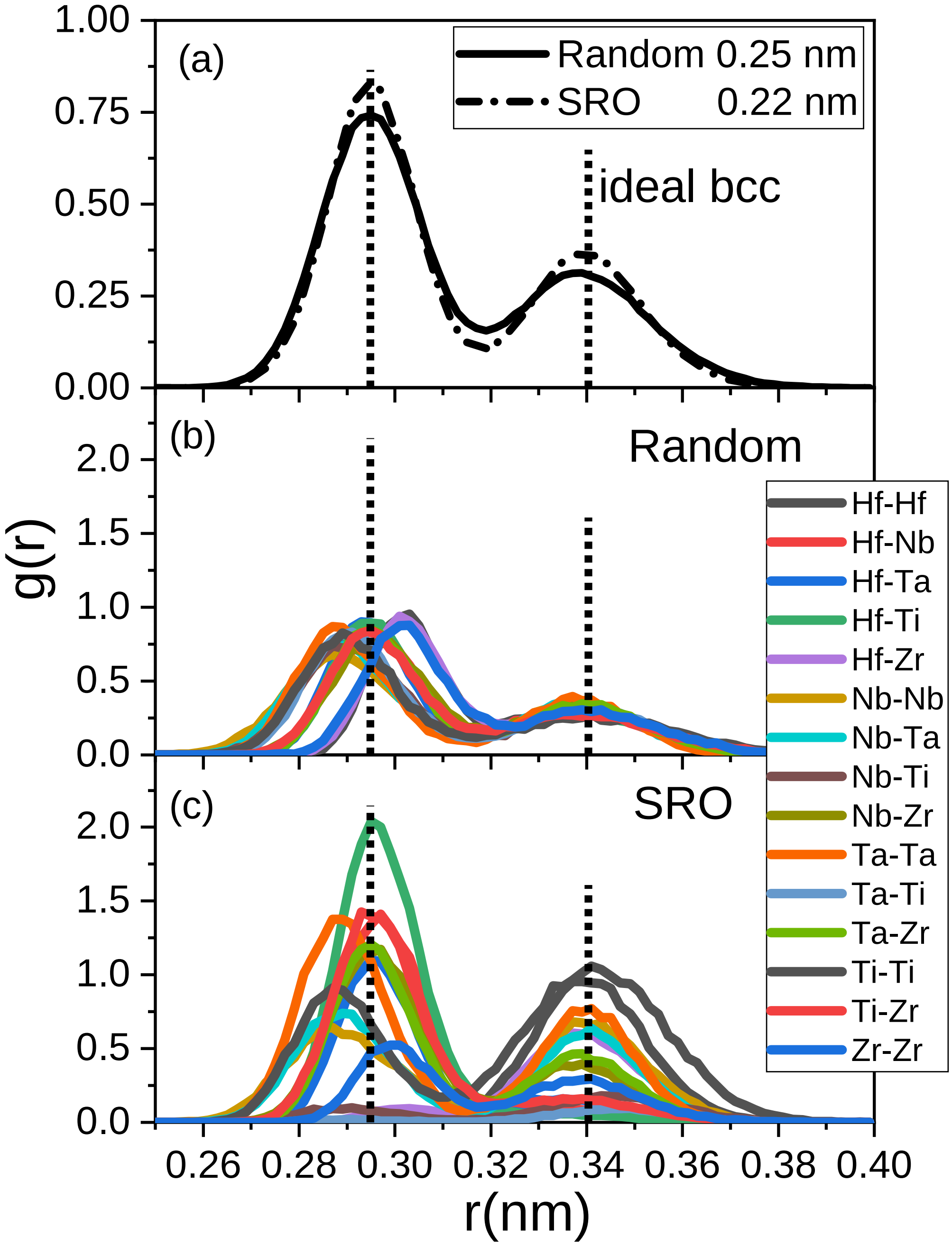

FIG. S1: (a) Pair correlation function $g(r)$ for the perfect crystal (vertical dashed lines), the random sample, and the SRO sample after 4.7e6 MC steps, after relaxation. In the perfect crystal used for the MC simulations all atoms sit on ideal lattice sites, independently of their chemical type. The width at half-maximum of the peaks is indicated in the legend. (b) and (c) present the partial pair correlation function for the random and the SRO sample, respectively.

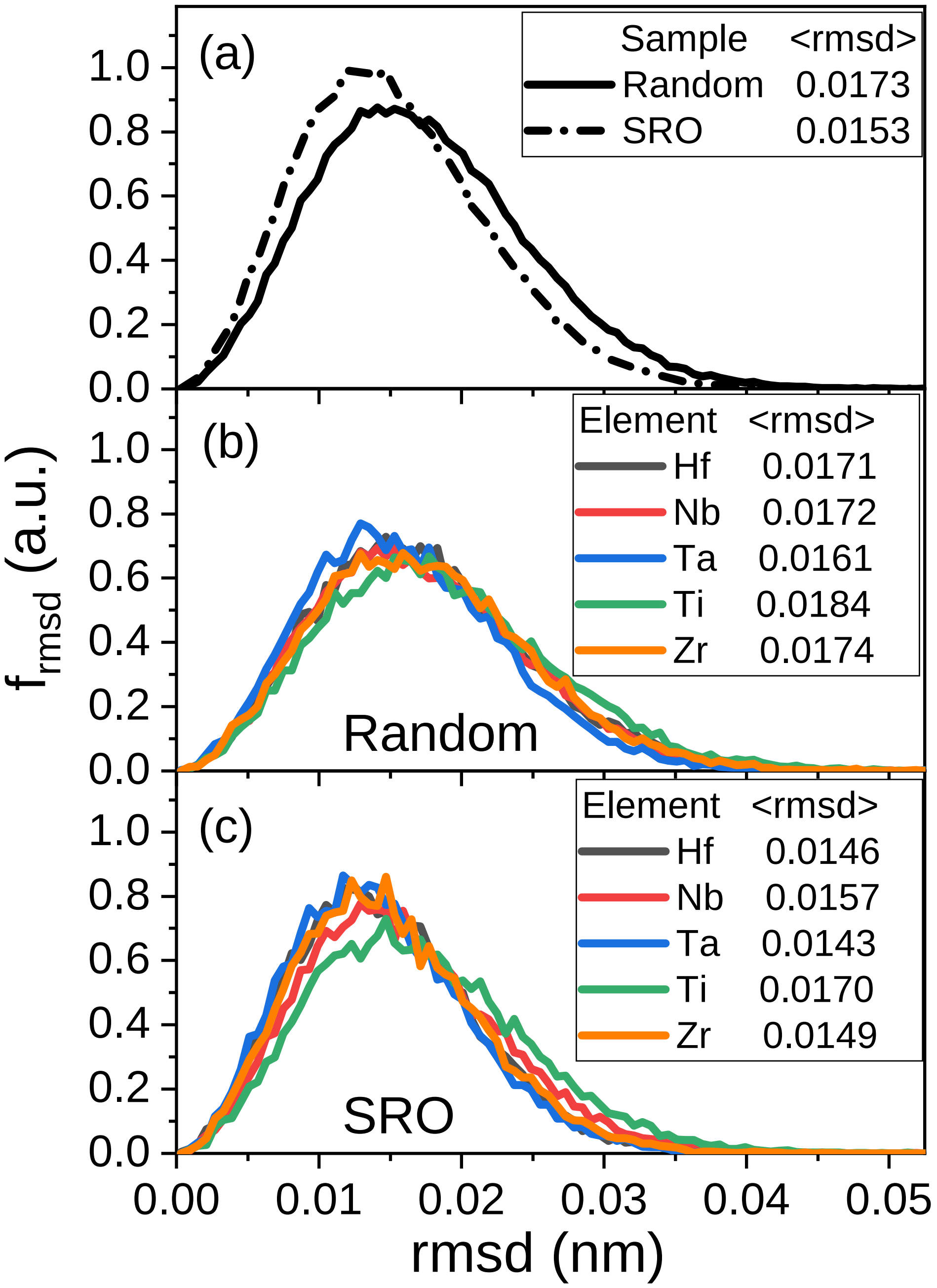

FIG. S2: Histograms of the atomic root mean square displacements (rmsd) with respect to the perfect crystal: (a) averaged over all atoms, (b) for the random sample, discriminated by chemical type, and (c) for the SRO sample, discriminated by chemical type. Insets indicate the corresponding mean values, in nm. The value for thermal displacements from the Debye model at 300 K is $\sim$ 0.007 nm.

MC steps	κ_{SRO} [W/(Km)]	volume fraction (c_i)			
		HfNb	HfTi	TaTa	TiZr
0	7.65E-01	2.34E-05	1.82E-05	1.04E-05	3.26E-05
1.0E+03	7.82E-01	6.77E-05	3.65E-05	3.77E-05	5.08E-05
5.0E+03	7.74E-01	6.38E-05	8.85E-05	3.91E-05	1.35E-04
1.0E+04	7.87E-01	1.68E-04	1.29E-04	1.55E-04	4.15E-04
5.0E+04	7.87E-01	1.53E-03	2.61E-03	4.12E-03	3.11E-03
1.0E+05	8.10E-01	5.65E-03	9.82E-03	5.31E-03	4.00E-03
5.0E+05	8.39E-01	1.28E-02	2.65E-02	6.26E-03	8.21E-03
1.0E+06	8.46E-01	1.46E-02	3.10E-02	6.30E-03	1.07E-02
2.0E+06	8.66E-01	1.55E-02	3.50E-02	6.30E-03	1.28E-02
4.7E+06	8.60E-01	1.58E-02	3.80E-02	6.10E-03	1.42E-02

TABLE S1: Heat conductivities, κ_{SRO}, and volume fractions of the different phases found in the short-range-ordered HEA after a certain number of MC steps.

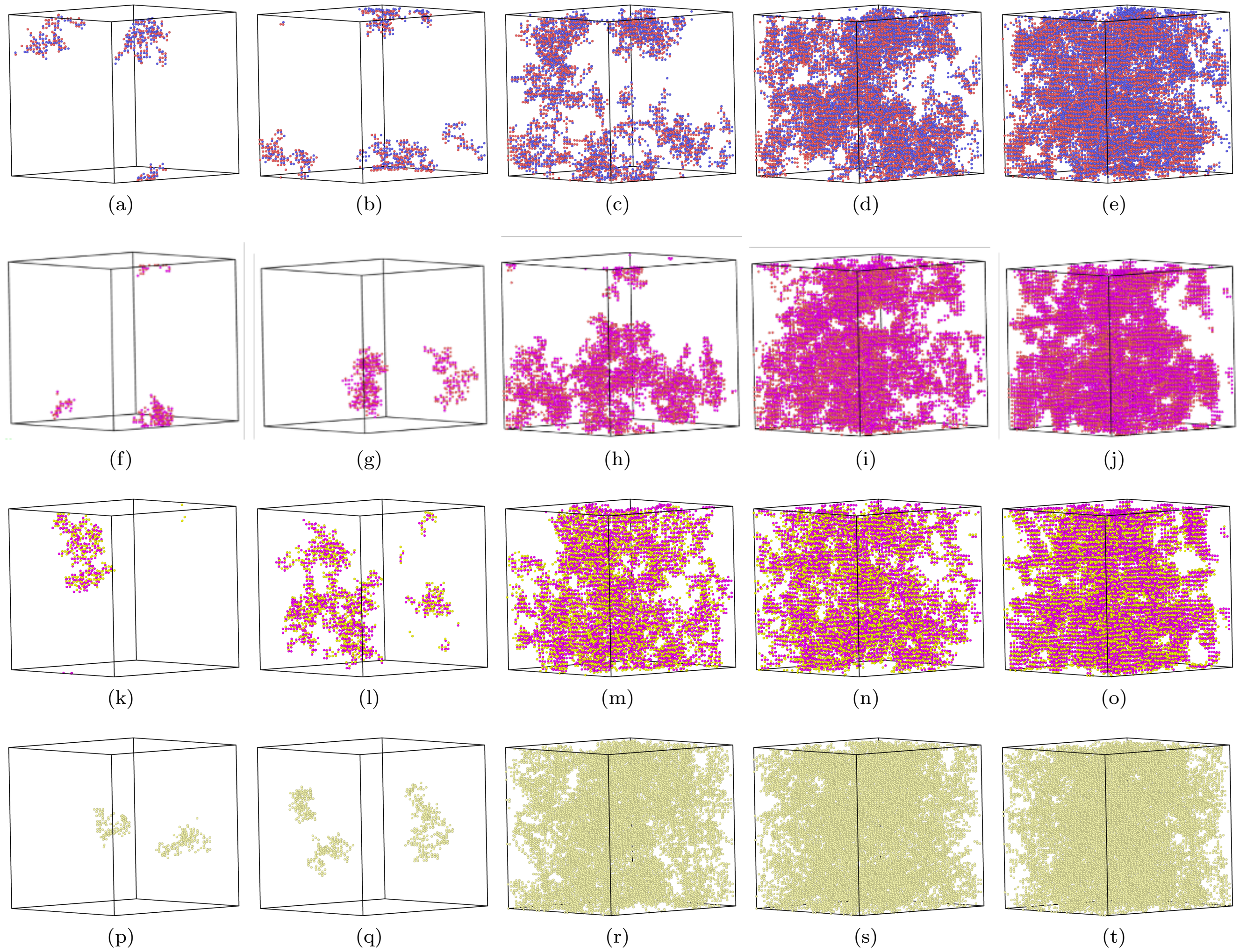

FIG. S3: Series of snapshots illustrating the presence of B2-structured clusters in samples with the SRO established after an increasing number of MC steps by column: 1 000, 10 000, 50 000, 100 000, 1 000 000; and the species by row: HfNb, HfTi, TiZr and Ta, respectively. Only the largest cluster is shown in each snapshot; due to the periodic boundary conditions it may appear disconnected at some steps, when is not.

Evolution of B2 HfTi clusters

Here we present additional characterization of the B2 HfTi clusters. Fig. S4 shows the evolution of the cluster size for all the HfTi clusters. In this figure, clusters identified in the sample are sorted in order of decreasing size. The figure shows that even in the random sample, a few HfTi clusters reaching sizes of > 100 atoms are present. These clusters grow during the MC evolution.

Figure S5 shows the histogram of the coordination for the atoms in the largest HfTi cluster, at different MC steps. A huge increase in the number of atoms with coordination 8 is observed at the percolation threshold obtained from the analysis in the main text. Still, the average coordination, shown in Figure S6 reaches only half the bulk coordination by the end of our simulation, showing a jump from 3 to 4 near the 'percolation' threshold.

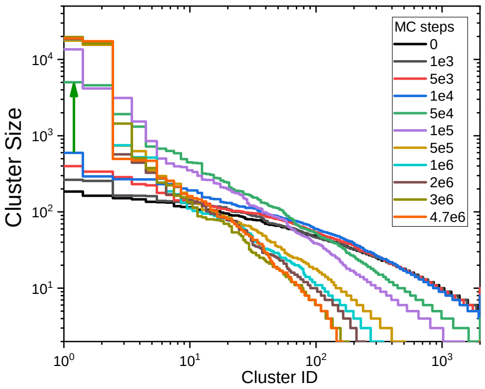

FIG. S4: Evolution of the HfTi cluster sizes (i.e., number of atoms contained) with the number of MC steps. Cluster ID identifies clusters in the order of descending size. The vertical arrow in the first bin indicates the large jump in the size of the largest cluster near the percolation threshold.

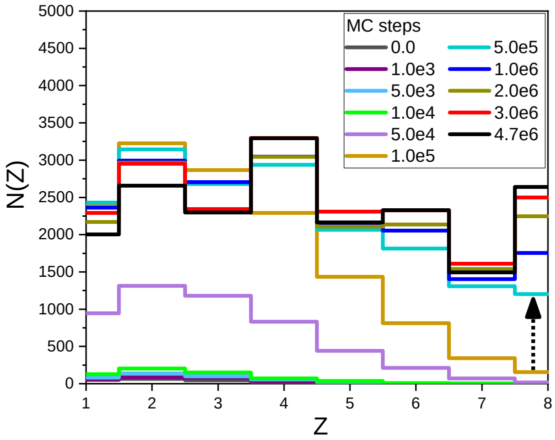

FIG. S5: Histograms of the coordination number Z for the largest cluster, at different MC steps. There is a large jump in the number of atoms with coordination $Z = 8$ (bulk coordination), from 1e5 to 5e5 MC steps, indicated by an arrow with dotted line. This is related to the percolation threshold discussed in the text.

Fig. S7a displays the fraction of sites occupied by the HfTi B2 phase, given as the number of those atoms in clusters larger than 8 atoms (for instance an atom and all the 8 nearest neighbors in the bcc lattice), normalized by the total

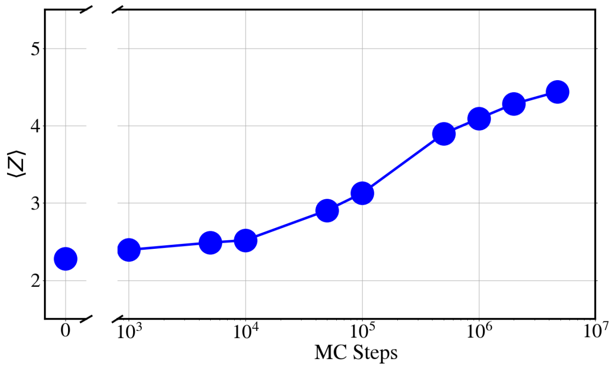

FIG. S6: Average coordination $\langle Z \rangle$ of the largest HfTi cluster vs. Monte Carlo steps.

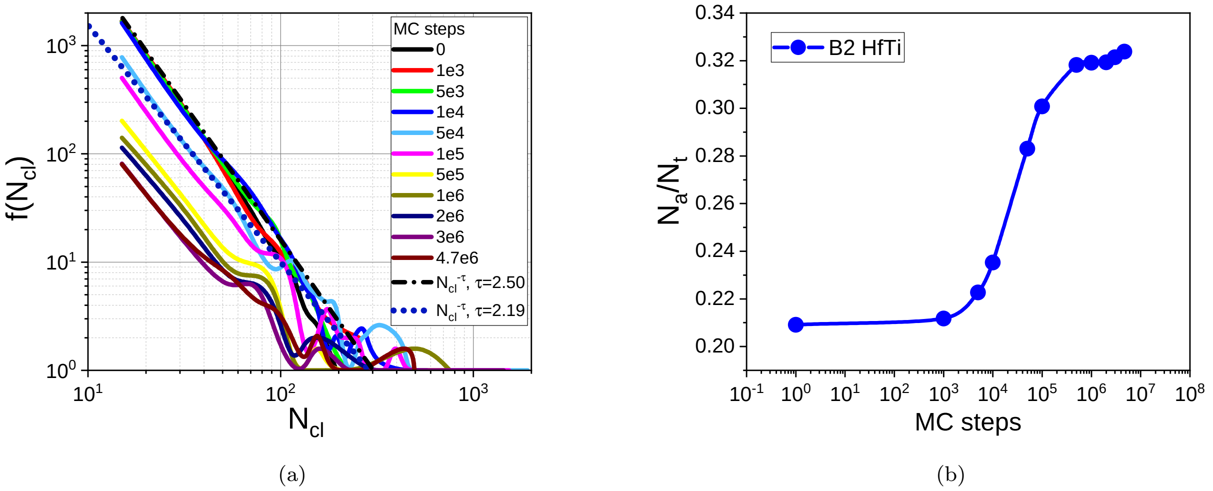

FIG. S7: Evolution of all B2 HfTi clusters larger than 8 atoms. (a) Cluster size ($N_{\rm cl}$) distribution for different MC steps. Power law fits indicate Fisher exponent τ for early times and near the percolation threshold. $\tau = 2.19$ for site percolation in a bcc lattice (b) Site fraction, given by the total number of atoms in all clusters, N_a, normalized by the total number of atoms in the lattice, N_t, versus MC steps, showing the percolation threshold near 1e5 MC steps.

number of atoms or sites in the sample. There is a clear jump which can be associated with the percolation threshold.

Fig. S7b quantifies the evolution of the distribution of cluster sizes with the number of MC steps for the example of HfTi B2 clusters. The power law size distribution is steep, with a Fisher exponent of 2.5. Note that the first four samples, for the earlier MC steps, shown overlap for cluster sizes less than 100. The distribution drops at small sizes and develops a tail at large sizes near the percolation onset. The Fisher exponent τ decreases and reaches the value expected for the bcc lattice site percolation of 2.19.

Fractal nature of SRO clusters

Since the number of atoms with high coordination seems to have a great impact on the behavior of the system, the surface-to-volume ratio could be relevant in our study. We use the *SurfaceMesh* tool in OVITO [1] to calculate it.

An analysis of the surface-to-volume fraction versus Monte Carlo steps is shown in Fig. S8. The fraction increases with MC steps, as the cluster has an initial growth, but after 1e5 steps there is a decrease, coincident with the percolation threshold, which signals a decrease of S/V. This decrease does not necessarily imply that the cluster is more compact, given the simultaneous large cluster growth.

Figure S10 shows this analysis, but we use spheres instead of cubic volumes as a simple approximation to the

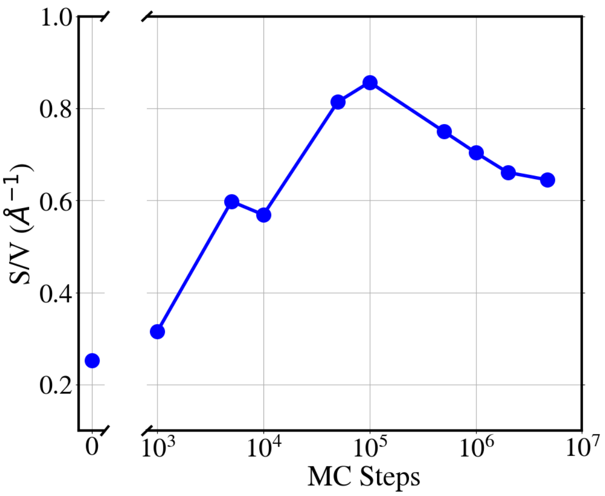

FIG. S8: Surface-to-volume ratio of the largest HfTi cluster vs. Monte Carlo steps.

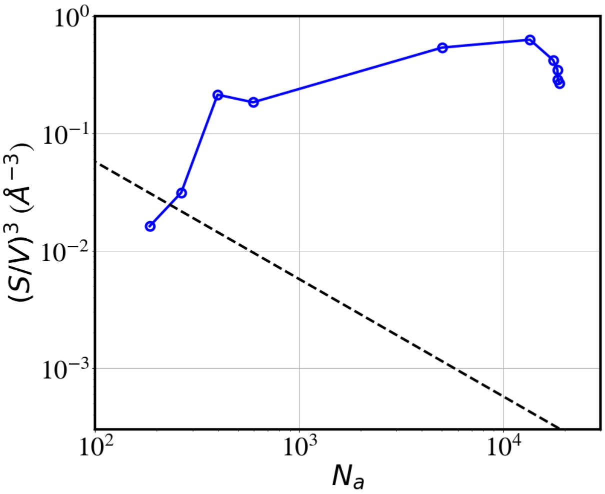

FIG. S9: Surface-to-volume ratio vs. number of atoms in the cluster, for the B2 HfTi largest B2 HfTi cluster at each MC step in Fig. S8. The dashed line shows the relation expected for an ideal spherical cluster.

analysis. N_c is equal to the ratio between the volume of the cluster and of a sphere of radius r, with the volume of the cluster calculated using the OVITO *SurfaceMesh* tool [2], with a virtual probe sphere of radius r. Then, the fractal dimension D_f can be calculated according to the following expression [3]:

$$N_c = k_f \cdot r^{-D_f},$$

where k_f is a constant.

Figure S11 shows a decrease in the evolution of D_f parameter with Monte Carlo steps, falling below 2 after the percolation threshold, and signaling the complex irregular growth of the percolating cluster. We note that irregular B2 clusters in a NbMoTa alloy, similar to those observed here in Fig. S3 and lacking a compact volumetric shape, have been recently reported as a result of diffusive jumps in [4].

Following the discussion in Ref. [5] for the case of standard 3D random percolation, D_f of the largest cluster should be ~ 2 well before percolation, and ~ 3 well afterwards. In addition, D_f at the transition can be approximated from the hyperscaling relation $\tau = d/D_f + 1$ that links the dimension, in this case $d = 3$, with the Fishers exponent, τ. This expression gives a value of $D_f = 2.52$ (using $\tau = 2.19$). In our simulations, we obtain $D_f \sim 2$ for the first point corresponding to the random system, as expected, and reaches values ~ 2.4 before the transition, close to the expected value of ~ 2.52. However, for values of MC steps after the percolative transition, the expected values of D_f closer

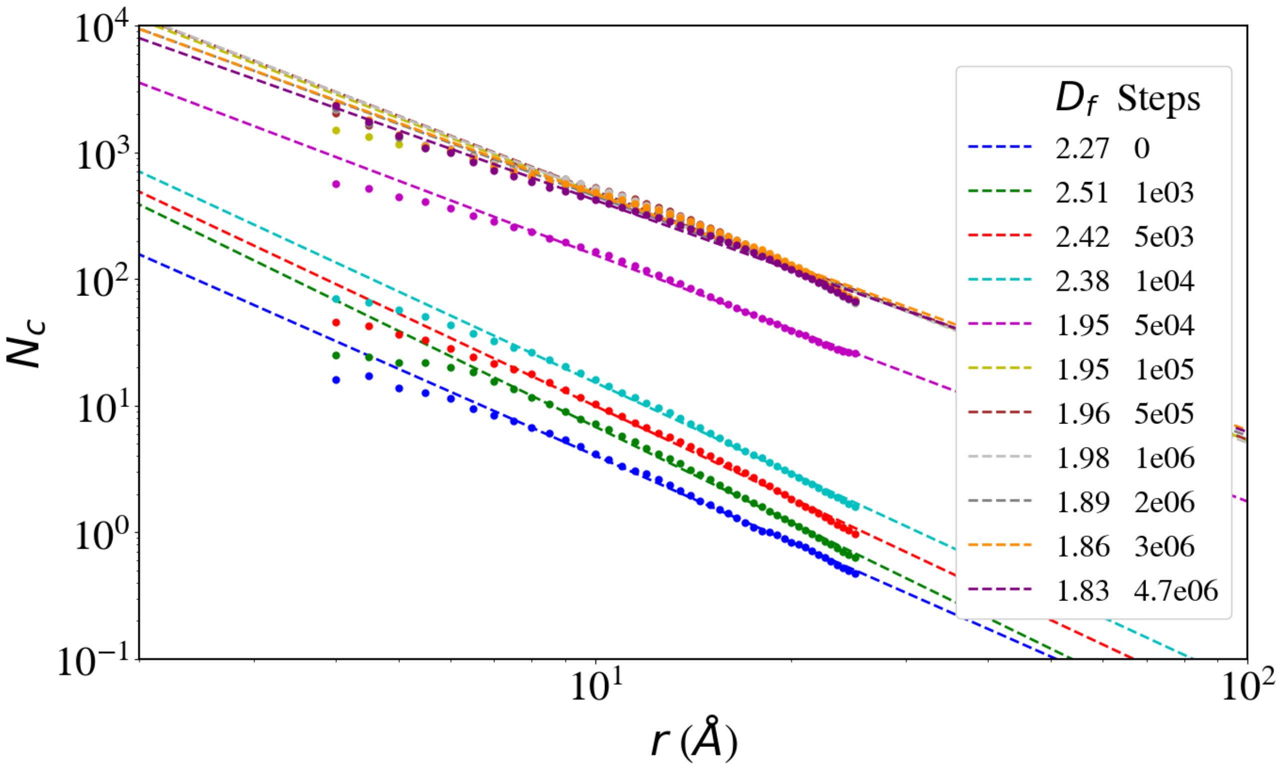

FIG. S10: Cluster volume N_c as defined in the text, for a given sampling size r, for various MC steps. For each step, the slope gives the fractal dimension D_f. Label indicates both D_f and the corresponding MC step.

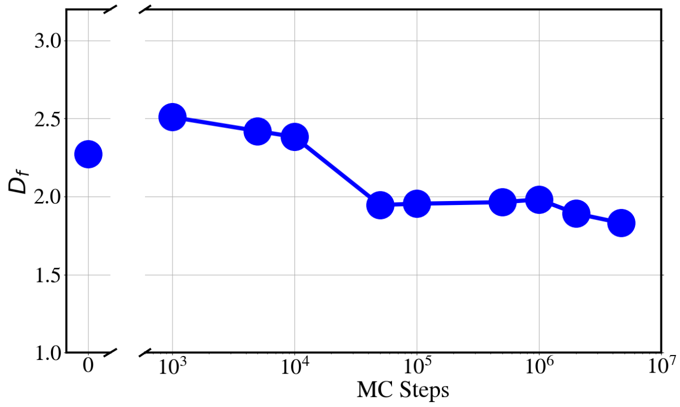

FIG. S11: Fractal dimension D_f vs. Monte Carlo step.

to 3 are not reproduced here. Instead, D_f estimations give values around 2 or even lower. This can be considered a consequence of the Kawasaki MC exchange process, which provides the main difference with the classical percolation model.

Thermal conductivity: analytical models using elastic constants

Table S2 includes values of thermal conductivity estimates from elastic constants, including all parameters required for those calculations, which follows equations collected in Ref. [6]. Elastic constants taken from MD simulations with the same potential, from Ref. [7], which agree very well with ab-initio simulation results [8]. Table S2 also

includes values of κ estimates for two samples studied in Ref. [6] with similar composition to the HEA study here for comparison.

The Cahill model [9] provides a lower limit estimate to the thermal conductivity, the mixed model gives phonon thermal conductivity [10], and the Slack model [11] would give a rough upper limit to the total conductivity, which for this alloy also includes an electronic component. We include results for two other similar alloys, and note that our results for the Slack and mixed models differ from those presented in Ref. [6], despite other quantities being the same.

These analytical models are for an 'average' material, since they only consider global elastic properties, which in this case are not significantly accepted by SRO. They allow for a fast estimate of the thermal conductivity and comparison with other publications which employ them, but MD results are only close to the minimum conductivity model, and differ significantly from the other two models, pointing to the need for improvements.

–	Random	SRO	MoNbTaTiZr	HfNbVTiZr
v_l [km/s]	4.32	4.39	4.25	4.05
v_s [km/s]	2.03	2.08	1.84	1.74
v_m [km/s]	2.28	2.34	2.07	1.96
θ_D [K]	252	258	247	231
γ_a	2.20	2.17	2.46	2.48
κ_{min} [W/(Km)]	0.64	0.65	0.7	0.65
κ_{mix} [W/(Km)]	5.95	6.56	3.54	2.63
κ_S [W/(Km)]	6.8	7.48	4.09	3.04
κ_{MD} [W/(Km)]	0.78	0.86		

TABLE S2: Parameters calculated from the elastic constants reported in Ref. [7], for the random sample and the SRO sample, after 1e7 steps. Longitudinal sound velocity, v_l, shear sound velocity, v_s, mean sound velocity, v_m, Debye temperature, θ_D, and Grüneisen parameter, γ_a. Thermal conductivities at 300 K: minimum conductivity [9], mixed model [10], Slack model [11], and MD result, κ_{MD}. Average mass is the same for random and SRO HEA, $M_a = 118.4$ g/mol, and atomic volume was assumed to be the same, $V_{at} = 2a_o^{-3} = 19.8$ Å^3. Results for two other similar alloys are also provided, using the elastic constants and atomic volumes from [6].

Thermal conductivity: analytical models assuming phase mixture

In order to describe the increase of the heat conductivity by chemical SRO, multi-phase models have to be used. The main text discuss a parallel model, Eq. 6, a serial model, Eq. 7, and a combination of both, Eq. 8. They allow to include several relevant phases and describe the MD results extremely well. Here we describe alternative composite material models, which can add complexity at the price of reducing the SRO HEA to a material with only two phases: the random HEA and a B2-ordered HfTi alloy with a volume fraction c. Both the serial and parallel model are recast here for this case with only two phases. In all these cases, the percolating cluster provides a fast pathway for heat conduction.

In the simple parallel model for binary compounds, following Eq. 6, the effective heat conductivity is given by:

$$k_{\mathrm{mix}} = (1-c)k_{\mathrm{HEA}} + ck_{\mathrm{Hf-Ti}},$$

where $k_{\mathrm{HEA}} = 0.76$ W/(Km) is the heat conductivity of the random HEA and $k_{\mathrm{Hf-Ti}} = 2.66$ W/(Km) is that of a HfTi crystal, as determined by our calculations. This mixing law describes a material in which the heat flux passes through either of the two media, and gives an upper limit on the heat conduction in a composite material. On the other hand, the serial model, Eq. 7 of the main text, gives a lower limit for conductivity in this binary-phase model. More refined models are available that make further assumptions on the heat transport pathway in the composite [12–14].

Fig. S12 shows the conductivity for this simplified binary compound, assuming that the phase fraction of B2 HfTi is given by the largest cluster, with volume fractions obtained with *SurfaceMesh* in OVITO [1]. The models shown are: Parallel model (PM, Eq. 6), Serial model (SM, Eq. 7), two variations of the Maxwell-Eucken model (ME1 and ME2), and the Effective Medium Theory (EMT). ME1 assumes isolated conductive precipitates in a matrix with less conductivity, while ME2 is the dual case, i.e., a conductive matrix with isolated, less conductive precipitates. EMT

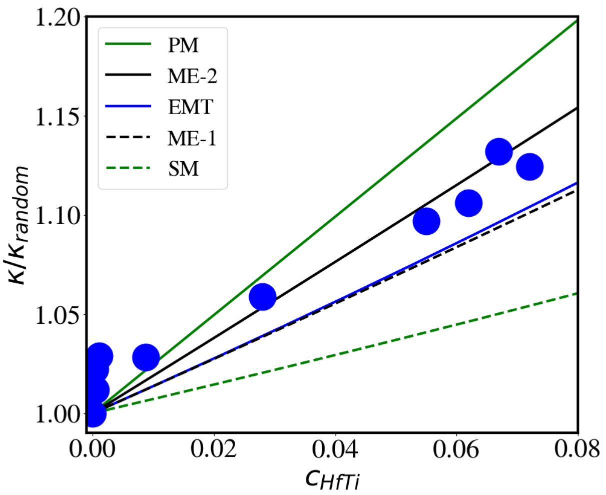

FIG. S12: MD thermal conductivity vs. HfTi volume fraction. The lines show different two-component continuous models: Parallel (PM) and Serial (SM) models in green (sometimes referred to as the Wiener bounds), the two forms of the Maxwell-Eucken model (ME-1, ME-2) in black, and the Effective Medium Theory (EMT) in blue [12].

assumes a uniform random distribution of the two phases. A detailed discussion of these models can be found in Refs. [12, 15].

MD results appear to be closer to the ME2 model in Fig. S12. However, this model only allows to consider two phases instead of the multiple phases involved. In addition, the ME2 model assumes that the matrix is more conductive, and that is the opposite of what happens in this case, where the random HEA matrix is less conductive.

Thermal conductivity versus Warren-Cowley parameters

It has been recently shown that heat conductivity and the Warren-Cowley parameters might be closely related [16].

Fig S13 shows the WC parameter of HfTi, $\alpha_{\rm HfTi}$, versus MC steps, and Fig. S14 shows the WC parameter versus the corresponding fraction of HfTi. We have found a dependence of the thermal conductivity on the SRO cluster fractions. HfTi is the dominant cluster, but other SRO clusters also play a role. However, one can expect a rough dependence of the thermal conductivity on the ‘dominant’ WC parameter, as shown in Fig. S15. This is similar to the recent simulation results for an fcc HEA [16].

The linear dependence of the heat conductivity on $-\alpha_{\rm HfTi}$ in Fig. S15, is reasonable, since this is an indication of the contribution of the largest SRO clusters. However, we consider that our description based on contributions from all SRO clusters instead of a single cluster is more appropriate and general.

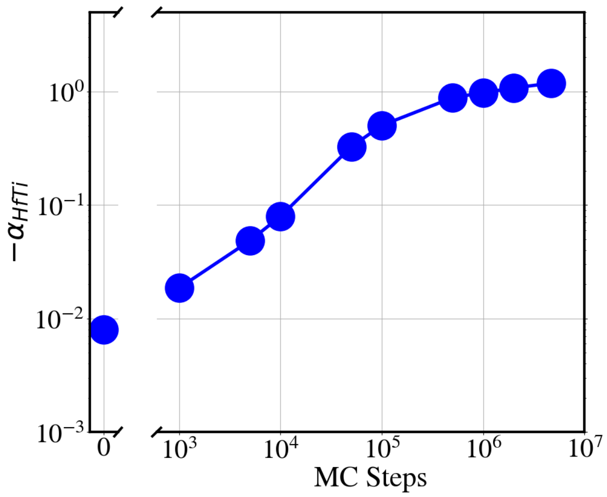

FIG. S13: Evolution of the negative HfTi WC parameter, $-\alpha_{\mathrm{HfTi}}$, with the number of Monte Carlo steps.

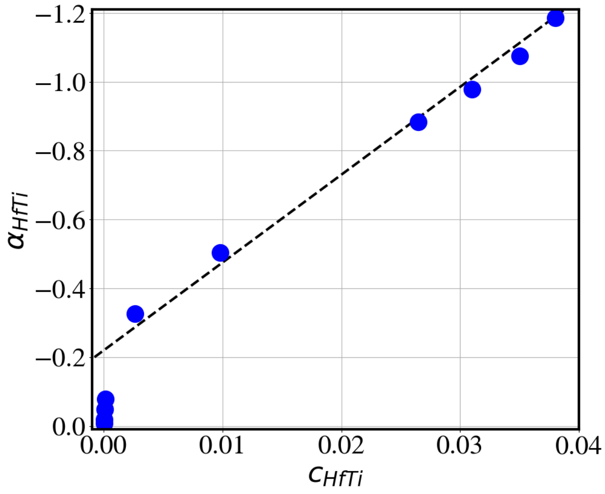

FIG. S14: HfTi WC parameter vs. HfTi volume fraction. The dashed line shows the linear fit $\alpha_{\mathrm{HfTi}} = -0.22 - 25.5c_{\mathrm{HfTi}}$.

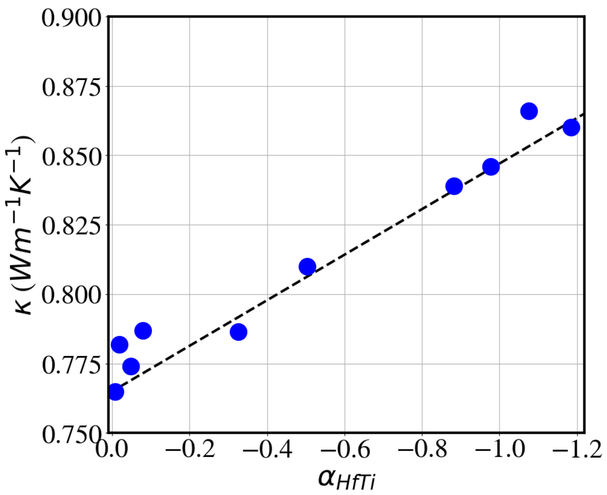

FIG. S15: MD Thermal conductivity vs. HfTi WC parameter. The dashed line shows the linear fit $\kappa = 0.765 - 0.082\alpha_{\mathrm{HfTi}}$, giving κ in units of W/mK.

Comparison of results obtained from different potentials

Besides the EAM potential [17] used in the present study, a modified embedded atom model (MEAM) potential was developed for the HEA HfNbTaTiZr by Huang *et al.* [18, 19]. Fig. S16 compares the WC parameters obtained for these two potentials and compares them to the result of ab-initio calculations [17].

The temperatures at which the WC parameters were calculated differ slightly from each other: 100 K for EAM (this work), 300 K for MEAM [20] and 400 K for DFT [17]. However, differences between the results obtained for the two potentials and DFT are obvious. This points to difficulties in the design of an empirical interatomic potential that faithfully reproduces all aspects of the SRO in this HEA.

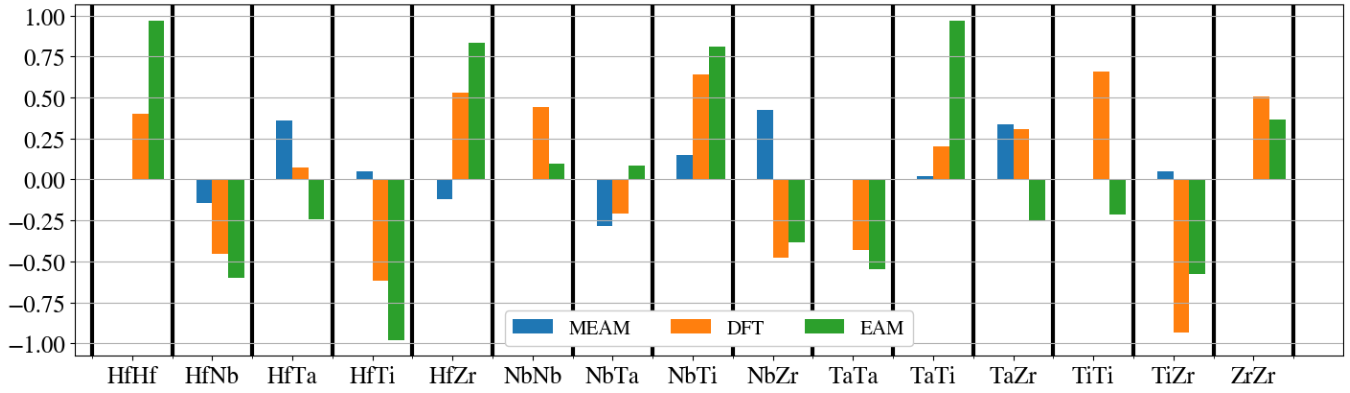

FIG. S16: Comparison of the Warren-Cowley parameters, obtained from DFT and from MD using the MEAM potential by Huang *et al.* [18, 19] and the EAM potential by Xu *et al.* [8]. Blue: MD data for the MEAM potential at 300 K reported by Aliaga *et al.* [20]; this reference does not include same-atom-type WC parameters. Orange: DFT data at 400 K reported by Xu *et al.* [17]. Red: results from our own MD simulations using the EAM potential at 100 K.

Evolution of potential energy during Monte Carlo simulation

Fig. S17 displays the evolution of the potential energy per atom in the sample with the number of Monte Carlo steps. It demonstrates that after 4.7e6 steps, the sample has nearly reached equilibrium.

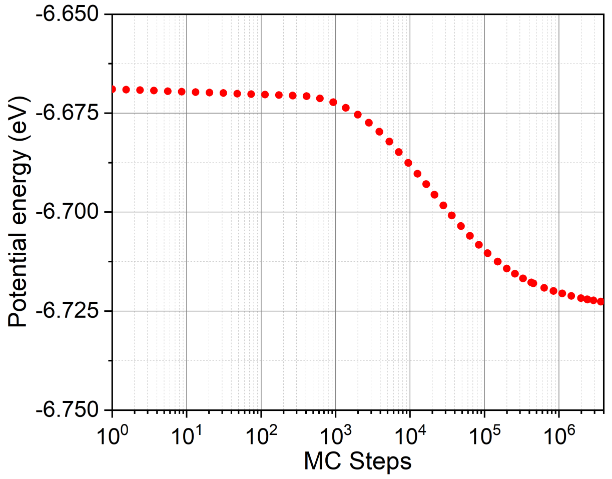

FIG. S17: Evolution of the potential energy per atom in the sample with the number of Monte Carlo steps.

[1] A. Stukowski, JOM **66**, 399 (2014).
[2] A. Stukowski, Model. Simul. Mater. Sci. Eng. **18**, 015012 (2010), *http://www.ovito.org/*.
[3] D. Giménez, R. Allmaras, E. Nater, and D. Huggins, Geoderma **77**, 19 (1997).
[4] B. Xing, T. J. Rupert, X. Pan, and P. Cao, Nature Communications **15**, 3879 (2024).
[5] D. Stauffer and A. Aharony, *Introduction to percolation theory* (Taylor & Francis, 2018).
[6] S. San, S. Hasan, P. Adhikari, and W.-Y. Ching, Metals **13**, 1953 (2023).
[7] I. Alabd Alhafez, O. R. Deluigi, D. Tramontina, N. Merkert, H. M. Urbassek, and E. M. Bringa, Scientific Reports **14**, 9112 (2024).
[8] S. Xu, W.-R. Jian, and I. J. Beyerlein, APL Materials **10**, 111107 (2022).
[9] D. G. Cahill, S. K. Watson, and R. O. Pohl, Phys. Rev. B **46**, 6131 (1992).
[10] E. S. Toberer, A. Zevalkink, and G. J. Snyder, J. Mater. Chem. **21**, 15843 (2011).
[11] D. T. Morelli and G. A. Slack, in *High thermal conductivity materials* (Springer, 2006), pp. 37–68.
[12] J. K. Carson, S. J. Lovatt, D. J. Tanner, and A. C. Cleland, International Journal of Heat and Mass Transfer **48**, 2150 (2005).
[13] Z. Hashin and S. Shtrikman, Journal of Applied Physics **33**, 3125 (1962).
[14] S. Kirkpatrick, Rev. Mod. Phys. **45**, 574 (1973).
[15] J. Wang, J. K. Carson, M. F. North, and D. J. Cleland, International Journal of Heat and Mass Transfer **49**, 3075 (2006).
[16] M. A. Al Hasan, S. Shin, and P. K. Liaw, Computational Materials Science **239**, 112980 (2024).
[17] K. Xun, B. Zhang, Q. Wang, Z. Zhang, J. Ding, and E. Ma, Journal of Materials Science & Technology **135**, 221 (2023).
[18] X. Huang, L. Liu, X. Duan, W. Liao, J. Huang, H. Sun, and C. Yu, Materials & Design **202**, 109560 (2021).
[19] X. Huang, L. Liu, W. Liao, J. Huang, H. Sun, and C. Yu, Acta Metallurgica Sinica (English Letters) **34**, 1546 (2021).
[20] L. C. R. Aliaga, A. M. Barboza, L. M. de Couto, and I. N. Bastos, in *High Entropy Alloys – Composition and Microstructure Design*, edited by Y. Yin, H. Huang, M. Zhang, and L. Zhou (IntechOpen, Rijeka, 2024), chap. 3.
